# Mild photothermal/radiation therapy potentiates ferroptosis effect for ablation of breast cancer via MRI/PA imaging guided all-in-one strategy

**DOI:** 10.1186/s12951-023-01910-6

**Published:** 2023-05-08

**Authors:** Zhe Zhang, Hsuan Lo, Xingyang Zhao, Wenya Li, Ke Wu, Fanchu Zeng, Shiying Li, Hongzan Sun

**Affiliations:** 1 Department of Radiology, Shengjing Hospital of China Medical University, Sanhao Street No. 36, Heping District, Shenyang, 110004 China; 2 Guangdong Cardiovascular Institute, Guangdong Provincial People’s Hospital, Guangdong Academy of Medical Sciences, Guangzhou, 510080 China; 3 Medical Research Institute, Guangdong Provincial People’s Hospital (Guangdong Academy of Medical Sciences), Southern Medical University, Guangzhou, 510080 China

**Keywords:** MR/PA imaging, Ternary metallic nanoparticles, Sensitized apoptosis, Photothermal/radiation therapy, Sensitized ferroptosis

## Abstract

**Background:**

Nanotheranostics advances anticancer management by providing therapeutic and diagnostic functions, that combine programmed cell death (PCD) initiation and imaging-guided treatment, thus increasing the efficacy of tumor ablation and efficiently fighting against cancer. However, mild photothermal/radiation therapy with imaging-guided precise mediating PCD in solid tumors, involving processes related to apoptosis and ferroptosis, enhanced the effect of breast cancer inhibition is not fully understood.

**Results:**

Herein, targeted peptide conjugated gold nano cages, iRGD-PEG/AuNCs@FePt NPs ternary metallic nanoparticles (Au@FePt NPs) were designed to achieve photoacoustic imaging (PAI)/Magnetic resonance imaging (MRI) guided synergistic therapy. Tumor-targeting Au@FePt forms reactive oxygen species (ROS), initiated by X-ray-induced dynamic therapy (XDT) in collaboration with photothermal therapy (PTT), inducing ferroptosis-augmented apoptosis to realize effective antitumor therapeutics. The relatively high photothermal conversion ability of Au@FePt increases the temperature in the tumor region and hastens Fenton-like processes to achieve enhanced synergistic therapy. Especially, RNA sequencing found Au@FePt inducting the apoptosis pathway in the transcriptome profile.

**Conclusion:**

Au@FePt combined XDT/PTT therapy activate apoptosis and ferroptosis related proteins in tumors to achieve breast cancer ablation in vitro and in vivo. PAI/MRI images demonstrated Au@FePt has real-time guidance for monitoring synergistic anti-cancer therapy effect. Therefore, we have provided a multifunctional nanotheranostics modality for tumor inhibition and cancer management with high efficacy and limited side effects.

**Graphical Abstract:**

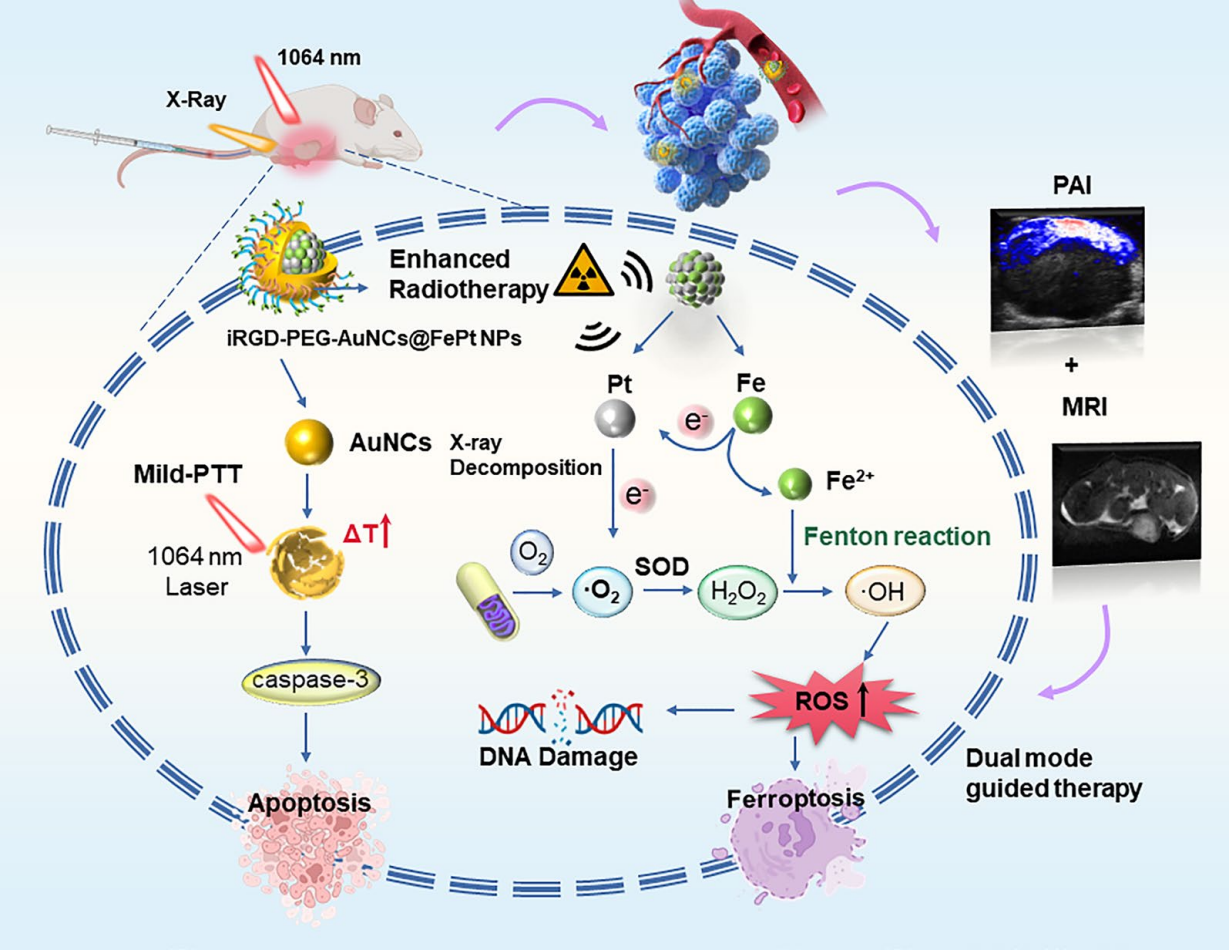

**Supplementary Information:**

The online version contains supplementary material available at 10.1186/s12951-023-01910-6.

## Introduction

Breast cancer is the most common cancer that occurs in women, with high aggressiveness characteristics, and most causes cancer-related death [1]. Considering the aggressive phenotype and worse prognosis, studies were committed to exploring the molecular biomarkers, diagnostic, monitoring, and predictive approaches to improve treatment for breast cancer [2]. Meanwhile, when combined with mammography, integrated contrast agents with highly sensitively targeted tumor properties used in magnetic resonance imaging (MRI) [3] can provide higher soft-tissue image quality, which is critical for screening and diagnosis in research and clinic [2]. Thus, the theranostics platform, as a newly emerging approach that combines structurally with drug delivery systems and targeted imaging agents [4] at the nano-size or molecular level [5], was considerable in providing beneficial diagnosis and therapy [6].

Additionally, recent advances in nanomedicine design based on regulated programmed cell death (PCD) have allowed us to elevate the therapeutic efficiency of tumors by promoting the apoptotic sensitivity in cancer cells [7]. As its application expanded, the fascinating synergistic tumor nanotherapeutics integrated multi-aspect coordination effects, including photodynamic therapy (PDT), photothermal therapy (PTT) [8–12], and chemodynamic therapy (CDT), etc. [13]. CDT, as an on-demand advanced therapeutic strategy, adequately diminishes cancer cells via catalytically releasing hydroxyl radical (·OH) within tumors. Nonetheless, those therapeutic strategies’ bio-application is limited by insufficient hydrogen peroxide (H_2_O_2_) levels and antioxidants [14, 15]. A concept of “nanocatalytic medicine” is proposed via integrating iron-based and non-iron-based nano catalysts [14, 16], such as ferumoxytol as iron oxide nanoparticles [17, 18], Cu2–xS and MnO_2_ for T1-weighted magnetic resonance (MR) imaging-guided CDT [16], generating ·OH in tumor via initiating catalytic reactions and depleting protective reductants. Although CDT/PDT-triggered apoptosis can provoke the anti-tumor response, the selectivity and efficiency of imaging-guided therapy are still impeded by off-target toxicity and the low tumor retention of therapeutic agents. Various nanoparticles have been extensively explored as ideal photothermal agents (PTAs) in the second near-infrared (NIR-II, 1000–1700 nm) window [19]. Therein, Au nanomaterials (e.g., gold nanoclusters and gold nanostars) with unique absorption properties can be assembled in highly selective and sensitive sensing theranostic platforms to provide photoacoustic (PA) imaging [20]. Moreover, increasing studies have found that theranostic platforms can remarkably elevate the selectivity of tumors by triggering PCD using PDT or CDT towards cancer therapy, combining non-invasive imaging techniques to improve the therapeutic effect [21–23]. Nevertheless, few present materials with dual-mode MRI/NIR-II PA imaging characteristics and synergistic therapeutic effects with deep penetration toward solid breast tumors.

PCD is inducted by tightly controlled intracellular signal transduction pathways which have been classified into various types based on their signal dependency, such as apoptosis, necroptosis, pyroptosis, ferroptosis, etc. [24]. In past decade, ferroptosis, an iron- and reactive oxygen species (ROS)-dependent form of PCD [25, 26], has attracted much attention and exploration in the cancer therapeutic mechanism of various nano-theranostics agents [27]. Among most of the reported nanomaterials, FePt and Fe_3_O_4_ NPs can reach the tumor area via enhanced permeability and retention (EPR) targeting effect. Ferrous Fe ions (Fe^2+^) accumulate due to the low pH in tumor cells, initiating Fenton reaction that recruits the overexpressed hydrogen peroxide (H_2_O_2_) (50–100 μM) to generate cytotoxic ·OH [28]. Furthermore, various studies have explored the anti-tumorigenic role of ROS and approached improving the ROS-dependent PCD efficiency [29], especially by chemotherapy and radiotherapy (RT) with real-time imaging, alleging more precise tumor therapy [30]. On this basis, high-Z metals-based radiosensitizers such as Pt-based drugs, Au nanoparticles, and different lanthanide-doped conversion NPs have been proposed in preclinical phase I and II trials [31–34]. Further studies reported by Klein et al. developed Au–Fe_3_O_4_ nano snowmen by epitaxially growing Fe_3_O_4_ onto a pre-synthesized Au nanosphere [31, 34]. Plasmonic nanospheres combined with a reactive Fe_3_O_4_ surface may significantly enhance XDT’s effectiveness as a therapeutic tactics due to their sizeable photoelectric absorption coefficient and ability to induct the equal strain and electronic band structures [14, 16, 35, 36]. Thus, this study proposes to develop Au@FePt ternary metallic nanoplatforms as a multimodal imaging-guided theranostic strategy to address orthotopic breast tumor elimination and therapeutic effects monitoring, which has great potential for preclinical translation.

**Scheme 1:**
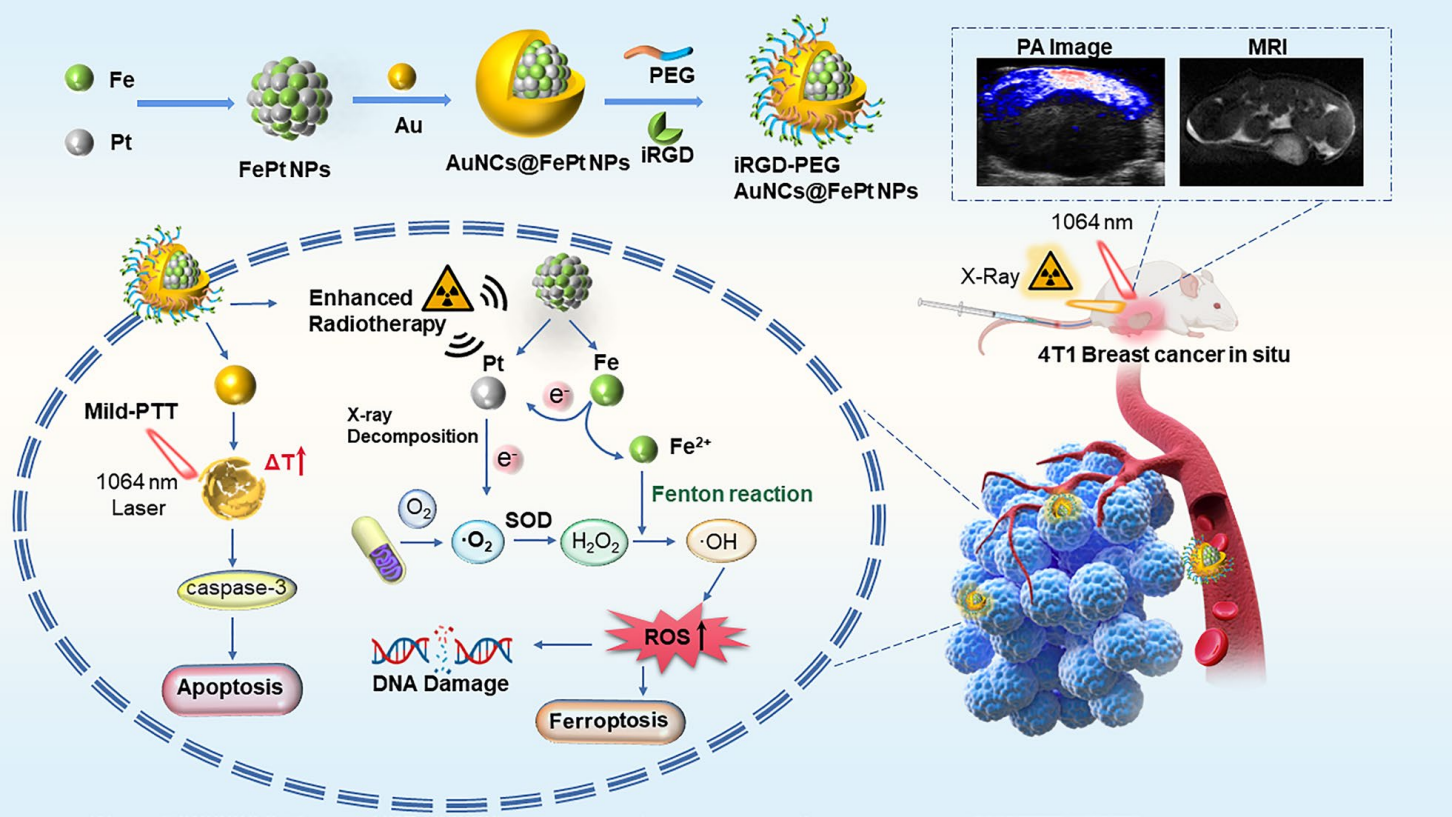
Facilely synthesis of Au@FePt ternary metallic NPs for dual-modal MRI/PA imaging with synergetic therapy

Specifically, we constructed a NIR-II mediated optimal and straightforward Au@FePt ternary metallic theranostics platform for targeted synergistic therapy toward orthotopic breast cancer. As depicted in Scheme 1, an Au@FePt ternary metallic nanoparticles (Au@FePt NPs) formulation achieved via a galvanic reaction to encapsulate microenvironment responsive FePt into a localized surface plasmonic resonance AuNCs. After tail injection, Au@FePt NPs provide NIR-II PA imaging and MRI (longitudinal relaxation time, T1) contrast images, visualizing deep tumors in a high resolution to monitor therapeutic efficiency. On the one hand, Au@FePt NPs can covalently bind intracellular GSH by the Au–S bond as well as participate in low-temperature PDT and CDT, and then using Pt conducts radiation therapy, thus specifically reducing tumor size. More importantly, RNA-sequencing suggested more insights into the mechanism of breast cancer of photothermal/radiation synergetic therapy induced by Au@FePt NPs. Therefore, this Au-FePt ternary metallic theranostics platform provided dual-modal MRI/PA imaging guidance for synergetic therapy through the precise apoptosis/ferroptosis effects in the orthotopic breast cancer model.

## Material and methods

### Materials

Chloroauric acid hydrate (HAuCl_4_·4H_2_O), sodium borohydride (NaBH_4_), sodium citrate silver nitrate (AgNO_3_), hydroxylamine hydrochloride (NH_2_OH·HCl), hydrochloric acid HCl (37%) and dimethyl sulfoxide (DMSO) were purchased from Sigma Aldrich. Poly (ethylene glycol) monomethyl ether thiol (SH-mPEG_2000_, MW ≈ 2000) was purchased from Laysan Bio (Arab, AL). DMSO and chloroform were obtained from TCI.

### Synthesis of FePt NPs

The FePt NPs were synthesized via a thermo-reduction standard protocol [37]. In brief, 290 mg Pt(acac)_2_ was mixed with 353 mg Fe(acac)_3_, 774 mg 1,2-hexadecandiol, 4 mL oleic acid, 4 mL oleylamine, and 4 mL dioctyl ether in a three-necked flask. Afterward, the mixture was heated to 100 °C (heating rate ~ 15 °C/min) under the protection of a gentle N_2_ flow. After 20 min, the reaction system was heated to 240 °C with a heating rate of 15 °C/min and maintained for 1 h before it was cooled to room temperature. Wash the product with chloroform and ethanol 3 times and stored it in chloroform for further modification.

### Synthesis of hydrophilic FePt NPs

50 mg of mPEG2000-SH was dissolved in 5 mL of chloroform by sonication, following the addition of 10 mg of FePt NPs dispersed in 5 mL of chloroform. The mixture was kept in a 55 °C water bath for 6 h. Thereafter, the product was washed with ethanol and ultrapure water successively to remove the physically adsorbed ligand on the particle surface. After freeze–drying, the powder was store in an N_2_ atmosphere for further use.

### Synthesis of targeted gold nanocage encapsulate FePt NPs (Au@FePt)

AuNCs were synthesized via the galvanic replacement reaction with silver NPs as the templates. Briefly, silver NPs were synthesized as previously reported [38–41]. AuNCs can be conveniently obtained by simply titrating with different portions of silver nanoparticles [42]. During the galvanic replacement reaction, FePt was added dropwise into the solution of silver NPs while titrating with 12 mL of (1 mM) HAuCl_4_ solution under heat and refluxed for 1 h. They were first modified by the addition of SH-PEG2000-NH_2_ (1 mg per 50 mL Au@FePt solution, LayBio) and stirring for 24 h to obtain PEG@Au@FePt. The molar ratio of SH-PEG2000-NH_2_ NPs and iRGD used for conjugation was 1:1.

### Characterization of Au@FePt

The UV–vis–NIR absorption spectra of the samples were measured with a spectrophotometer (Agilent 8453). The hydrodynamic size, zeta potential and stability of NPs were determined using a dynamic light scattering instrument (NanoSight NS500, Malvern Instruments Ltd, UK). The morphologies of Au@FePt and AuNCs were characterized using a transmission electron microscope (TEM, JEOL JEM-2010). Elements mapping was performed using a Themis Z aberration-corrected scanning transmission electron microscope (ac-STEM, FEI, USA). Energy-dispersive X-ray spectroscopy (EDX) spectra were acquired on an EX-250 EDX analyzer (HORIBA, Japan). X-ray diffraction (XRD) mapping was performed on an Ultima IV X-ray diffractometer (Rigaku Corporation, Japan) for the FePt NPs.

### Cell culture experiment

4T1 breast cancer cells (ATCC) were cultured in Dulbecco’s modified Eagle’s medium (DMEM, Thermo Fisher Scientific Inc.) supplemented with 10% fetal bovine serum (Thermo Fisher Scientific Inc.) and 1% penicillin–streptomycin (Thermo Fisher Scientific Inc.) in 37 °C humidified incubator with 5% CO_2_.

### Measurements of photothermal conversion efficiency

To measure the photothermal performance, 800 μL of Au@FePt dispersions with a series of Au@FePt concentrations (0, 5, 10, 20 ppm) in 1.5 mL tubes were irradiated by a NIR laser for 5 min (1064 nm, 1.2 W/cm^2^). The solution containing Au@FePt NPs was irradiated by a NIR laser (1.2 W/cm^2^) to its maximum value, and then the solution was cooled down to room temperature, and five cycles were repeated. All sample temperatures were monitored using a near-infrared thermal image (FLIR C3-X Systems Inc., USA). PBS was used as the negative control for all photothermal conversion efficiency measurements. The photothermal conversion efficacy (η) was calculated according to the approach as reported before, the details for the calculation are shown in Additional file 1.

### Free radical generation of Au@FePt nanoprobes

PBS (pH = 5.8) solutions of methylene blue (MB) (10 μg/mL) containing Au@FePt nanoprobes (Fe concentration: 100 μg/mL) and different concentrations of H_2_O_2_ (0, 2, 4, 8 and 10 mM) were incubated in a 37 °C aqueous bath for 30 min. Then, the obtained solutions were centrifuged at 6000 rpm for 20 min in ultrafilters (Millipore, MWCO = 30 kDa). Afterward, the absorbance changes of MB were measured. To investigate the influence of Fe concentration on the production of ·OH, the absorbance of MB and fluorescence spectra in PBS (pH = 5.8) treated with gradient Fe concentrations (0, 10, 20, 40, 80, 100 μg/mL) and H_2_O_2_ (10 mM) were measured.

### In vitro cytotoxicity

The standard CCK-8 assay was used to investigate the cytotoxic effect of Au@FePt, with and without NIR-II laser irradiation. In brief, 4T1 cells were seeded in 96-well plates at a density of 5 × 10^3^ cells per well and incubated with DMEM medium (150 μL) at 37 °C in 5% CO_2_ for 24 h. The culture medium was then replaced with 150 μL of freshly prepared culture medium containing the FePt at different Fe concentrations and Au@FePt NPs at different concentrations.

### Annexin V-FITC/PI double staining (flow cytometry)

For PTT, 4T1 cells were seeded in a 6-well plate 24 h before receiving various treatments (PBS, Au@FePt, Au@FePt + X-ray, Au@FePt + laser, and Au@FePt + laser + X-ray,) containing (100 μg/mL of AuNCs). The medium was replaced by a fresh medium before the NIR laser exposure (1064 nm, 1.2 W/cm^2^, 5 min), followed by incubation for 24 h. The cells were collected and washed with PBS three times then resuspended in 100 µL 1× annexin binding buffer. Harvested cells were counterstained with 100 µg/mL propidium iodide (PI) and FITC labeled annexin V for 15 min, and the stained cells were subjected to flow cytometry analysis (Beckman Coulter, CytoFLEX S).

### Intracellular Fe^2+^ and ROS fluorescence staining

4T1 cells were seeded into the co-focal dish (1 × 10^5^ cells per well) and subjected to co-incubation with Au@FePt nanoprobes, (AuNCs concentration: 50 ppm Fe concentration: 100 μg/mL) for 6 h with and without NIR-II laser irradiation (1064 nm). Afterward, the cells were stained with FerroOrange (Dojindo, Japan) and observed under confocal laser scanning microscopy (ZEISS LSM 780, Germany) using a 40× oil immersion objective lens.

4T1 cells were seeded into 12-well plates (1 × 10^5^ cells per well) and the cells were stained with DCFH-DA (Beyotime, China)then treated with Au@FePt nanoprobes (AuNCs concentration: 50 ppm, Fe concentration: 100 μg/mL) for 6 h with or without NIR-II laser irradiation (1064 nm) and X ray radiation (4 Gy) and observed under confocal laser scanning microscopy (ZEISS LSM 780, Germany) using a 40× oil immersion objective lens.

### Western blot analysis

Western Blot Analysis: for the combined effect of mild PTT with XDT/CDT, 4T1 cells were incubated with respective treatments (PBS, laser, Au@FePt + X ray, Au@FePt + laser and Au@FePt + laser + X ray; 50 ppm of AuNCs; 100 μg/mL of FePt). X ray radiation was applied using 4 Gy of X-ray irradiation. The medium was replaced by a fresh medium after 6 h, followed by NIR-II laser exposure. After 24 of hour incubation, total protein was extracted using lysis buffer. The protein concentrations were calibrated and determined via standard bicinchoninic acid assay. Electrophoresis was performed by SDS-PAGE with polyacrylamide gel and PVDF membrane blotting. The membranes were blocked by 5% BSA in TBST and incubated with primary antibodies overnight at 4 °C.

### Tumor model establishment

BALB/c femal mice (5–6 weeks of age, n = 4) were followed by subcutaneous injection of 10^7^ 4T1 breast cancer cells suspended in 50 μL DMEM on the inguinal gland of the mammary fat pad.

### In vivo PAI

The transducer LZ250 (21 MHz) would be applied to both in vitro and in vivo animal models for better orientation. Au@FePt (100 ppm for AuNCs and 2 mg/mL for Fept; 200 µL) was administered into the subject through the tail vein and imaged continuously for 2 days, every 2 h per image. The signal analysis detail is an illustration in Additional file 1.

### In vitro and in vivo MRI imaging

For the MRI switching experiment, the T1- and T2-relaxation rates of the Au@FePt nanoprobe dispersions in different concentrations were measured by a7T/310 NOVA animal MRI scanner (NOVA, Germany). Dispersions of Au@FePt nanoprobes at gradient concentrations were used for the evaluation of in vitro imaging performance under different modalities. For MRI, dispersions (Fe concentration: 0, 5, 10, 25, 50, and 100 ppm) were analyzed using a 7.0 T/310 MRI system (NOVA, Germany).

### In vivo tumor inhibition effect via mild PTT and XDT/CDT/PTT

The 4T1 tumor-bearing mice were randomly divided into 3 groups (n = 4) and intravenously injected with PBS, Au@FePt, Au@FePt + X-ray, Au@FePt + laser, and Au@FePt + laser + X-ray (5 mg/kg FePt, 30 mg/kg AuNCs, 200 μL), pending PTT treatments. Tumor regions were irradiated by the NIR-II laser 6 h after injection (1.2 W/cm^2^, 5 min × 2 times). X ray radiation was applied using 4 Gy of X-ray irradiation. The surface temperature was recorded using a thermal camera (FLIR C3-X Systems Inc., USA and quantified by the FLIR Thermal Studio software). Tumor volumes were calculated following a well-established formula: V = L × W × W× π/6 (π/6=0.52) where L and W stand for the length and width of the tumor, respectively. Besides, at the end of the experiment, those organs of mice were harvested to calculate the organ coefficient, whichwas calculated using the following formula: Organ coefficient = weight of the organ (mg)/body weight (g).

### Immunofluorescence staining and histopathological evaluation (H&E)

To further prove the RCD process involved ferroptosis, an immunofluorescence experiment on tumor tissue that had received various treatments was performed. After the sacrifice of the mice-bearing mice, the xenograft tumors from 5 individual groups, including PBS Au@FePt, Au@FePt + X-ray, Au@FePt + laser, and Au@FePt + laser + X-ray, were collected and fixed with 4% formaldehyde in PBS. The sampled tissues were then embedded in paraffin for further staining. The experiment details immunofluorescence staining of DCFH-DA and 4HNE were illustrated in Additional file 1.

### Statistical analysis

The data represent the mean ± SD from three independent experiments, each performed in three replicates. Statistical comparisons were carried out in GraphPad Prism 5 software GraphPad Software Inc.). Differences between two groups were calculated and tested by a student’s t-test. **P < 0.05 and *P < 0.01 were considered to be significant difference between groups.

## Results and discussions

### Preparation and characterization of FePt, AuNCs and Au@FePt NPs

**Fig. 1:**
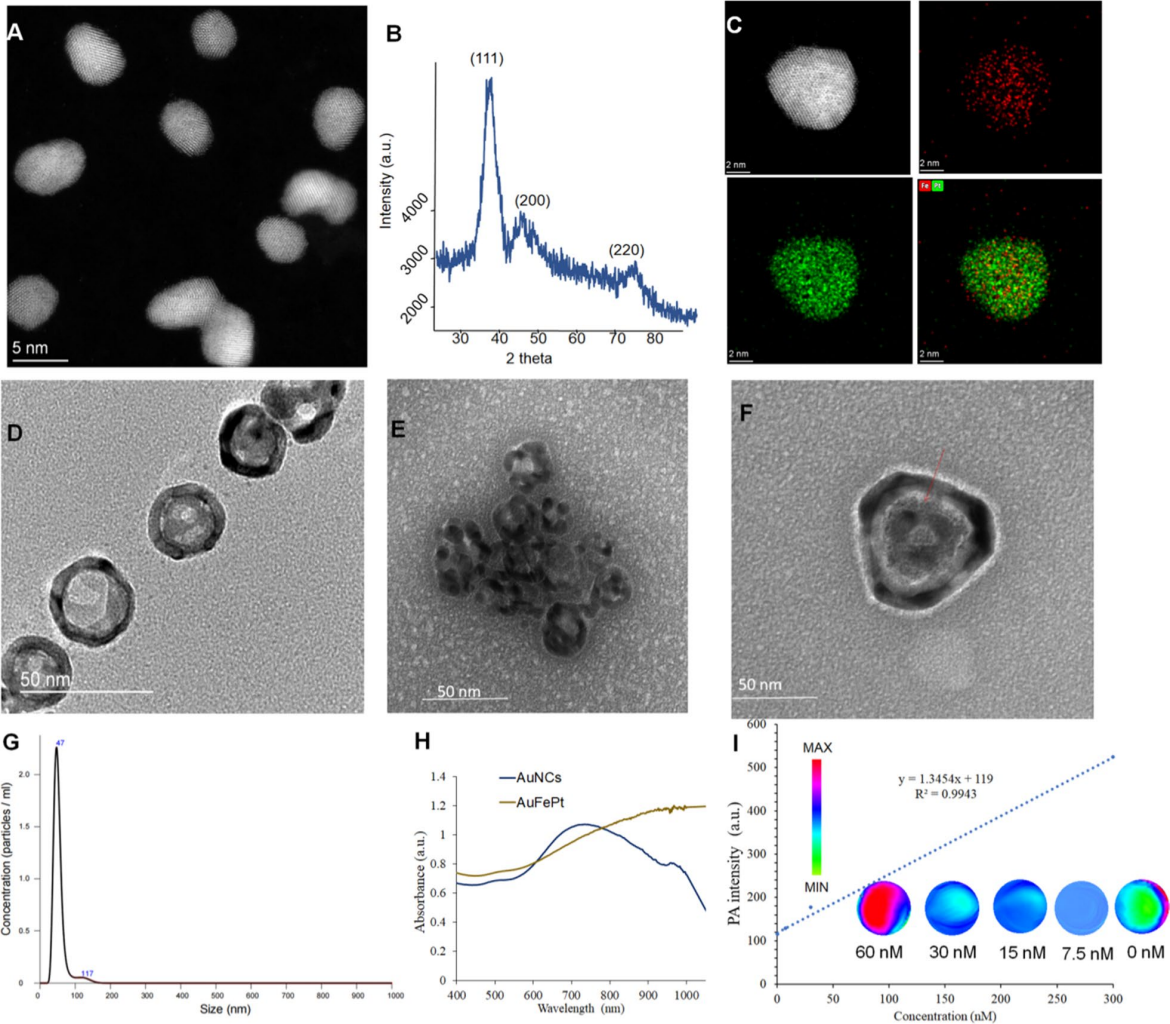
**A** The TEM images and **B** the powder X-ray diffraction (PXRD) analysis of FePt nanoparticles (scale bar = 5 nm). **C** The STEM-EDS elemental mapping images and **D** the TEM images of AuNCs nanoparticles. **E** The TEM images of Au@FePt nanoparticles. **F** The high resolution TEM of one gold nanocage loaded FePt. **G** Hydrodynamic size distribution of Au@FePt nanoparticles. **H** UV–Vis spectrum of Au@FePt and AuNCs nanoparticles. **I** PA intensity of Au@FePt NPs in different concentrations

The FePt and AuNCs NPs were synthesized using the literature method with modifications, and a series of experiments were conducted to determine the morphology of FePt, AuNCs and Au@FePt NPs, such as TEM, XRD and DLS. The morphologies revealed by high-resolution TEM (HRTEM) for FePt and Au@FePt were shown in (Fig. 1A) which shows the uniform shape of FePt nanoparticles, which were approximately 4 nm in size. Figure. 1B revealed that all three diffraction peaks were indexed to FePt. Elemental mapping of single and multiple FePt particles is shown in Fig. 1C which demonstrated Fe and Pt species presented in the nanoparticles. The TEM image of AuNCs is shown in Fig. 1D to determine the surface morphology of the cage’s existing hole to load small molecules and HRTEM images was shown in Additional file 1: Fig. S2. Figure 1E shows a high payload of FePt in AuNCs for dual-mode imaging as exogenous contrast agents. The intrinsic properties of Au@FePt NPs were investigated in HRTEM with the loading of FePt NPs revealing a morphology with a diameter of ~ 4 nm inside the hole of the AuNCs. The particle size and distribution by DLS of Au@FePt in Fig. 1G revealed that the size of Au@FePt slightly increased (approximately 40 nm). The absorption spectra of AuNCs and Au@FePt NPs are presented, indicating the encapsulation process did not affect the optical properties of AuNCs and FePt to serve as excellent photothermal agents. Besides, the absorption of Au@FePt NPs displayed a broad peak centered at 900 nm in pH 6.5 phosphate buffer solution (PBS) (Fig. 1H) which indicated the local surface plasmon resonance (LSPR) broadens the NIR absorption region. To further evaluate the homogeneity and dispersibility of Au@FePt in different pH buffers upon laser irradiation [43], the change in hydrodynamic radius of Au@FePt was recorded for 1 day as shown in Additional file 1: Fig. S3. The result suggests the Au@FePt nanoprobe could be a suitable candidate under normal physiological condition for drug delivery applications.

### Photoacoustic and photothermal properties of the Au@FePt NPs

**Fig. 2:**
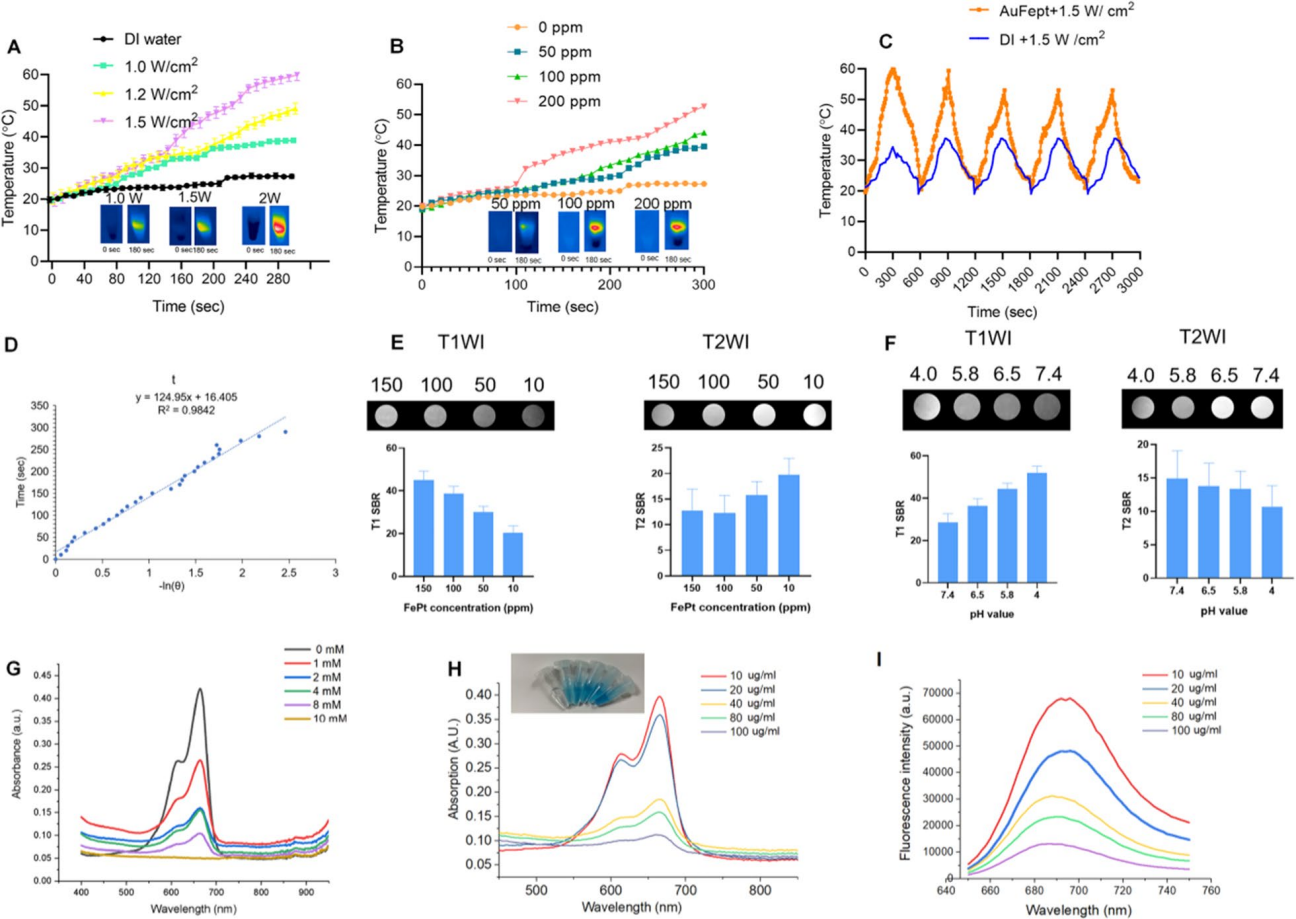
**A** Temperature elevation curves of Au@FePt NPs (100 ppm) under different laser powers. **B** Temperature changes curve of Au@FePt NPs (100 ppm) in different concentrations during 5 min laser irradiation at 1.2 W/cm^2^. **C** Multiple heating cycles of Au@FePt NPs (100 ppm) (orange plot) and DI water (blue plot). **D** The relationship between the first cooling time and the negative natural logarithm of the temperature-driving force was calculated from Au@FePt nanoparticles. **E** In vitro T1 and T2 signal-to-background (SBR) of Au@FePt in different concentration. **F** T1 and T2 SBR of Au@FePt nanoprobes incubated at various pH. **G** Methylene blue (MB) (10 μg/mL) degraded by ·OH generated from Au@FePt nanoprobes (Fe concentration: 100 µg/mL) and different concentrations of H_2_O_2_ (0, 2, 4, 8, and 10 mM). **H** MB absorbance after incubation with Au@FePt nanoprobes on the production of ·OH, with gradient Fe concentrations (0, 10, 20, 40, 80, 100 μg/mL) and H_2_O_2_ (10 mM) was measured. **I** MB fluorescence spectra after incubation with various concentrations of Au@FePt nanoprobes (0, 10, 20, 40, 80, 100 μg/mL) and H_2_O_2_ (10 mM) were measured

As shown in Fig. 1I, the Au@FePt NPs exhibit intensive PAI signals upon irradiation at 1064 nm captured by the Vevo 2100 animal PAI system, and their PA signals elevate linearly with the Au@FePt concentrations. The high PAI sensitivity was mainly due to the conspicuous absorption of Au@FePt NPs at 1064 nm. Photothermal-heating curves of Au@FePt NPs and corresponding thermal images were displayed to reveal that Au@FePt NPs are feasible for photothermal agents [44]. Furthermore, after 1064 nm laser irradiation, the temperature elevation of Au@FePt NPs in aqueous solution increased in a laser power-dependent manner (from 1.0 to 1.5 W/cm^2^), as shown in Fig. 2A. As shown in Fig. 2B, the temperature for Au@FePt NPs increased considerably with the increase in AuNCs’ concentration. In contrast, the temperature of the control group’s deionized (DI) water showed negligible changes at 1.2 W/cm^2^, which demonstrated that the Au@FePt NPs could convert NIR-II light energy into thermal energy adequately [45]. In addition, just a negligible change was noticed after three repeated heating cycles upon 1064 nm laser on/off (1.5 W/cm^2^) of Au@FePt NPs as indicated in Fig. 2C. Significantly, the AuNCs contributed excellent photothermal conversion properties to to Au@FePt NPs which affords good evidence for in vivo PTT experiments. The photothermal conversion efficiency value (η) was 31.3% as shown in Fig. 2D which was a bit higher than previously reported nanoparticles [46]. These results showed that this Au@FePt nanoprobe has significantly high photothermal conversion ability and is an ideal photothermal agent for dual imaging guided mild PTT combined with CDT. Au@FePt nanoprobes containing 1 mg Fe were dispersed in 1 mL HNO_3_ solution (pH = 5.8, 7.4, with or without laser) in a dialysis tubing bag and the filtrates were collected for Fe concentration quantification with ICP-OES with at 24-h intervals. The results indicated ~ 18.9%, ~ 8.2% (with laser irradiation) ~ 6.2% and 2.4% (without laser irradiation) Fe release of Au@FePt nanoparticles under various pH conditions with and without laser for 24 h (Additional file 1: Fig. S4).

### Responsive MRI signal of the Au@FePt NPs

The results from Fig. 2E showed that the MRI signal was significantly more sensitive for detecting injected phantoms which are a positive T1-weighted imaging (T1WI) enhancer when releasing Fe^2+^. Moreover, the paramagnetic Fe atoms that remain in the undissociated FePt probes are excellent negative T2WI contrast agents (Fig. 2E), comparable to a recent report, while the released Fe^2+^ can be acted as a positive T1 WI contrast agent. Based on 7.0 T MRI data, the T2-weighted relaxation rate of Au@FePt was 79.361 s^−1^ and the T1-weighted relaxation rate was 4.027 s^−1^, emphasizing the competence of the Au@FePt nanoprobe as T1/T2-weighted dual-mode MRI contrast agent, raising the possibility of a method to precisely identify the tumor region to be detected [37]. In terms of the MRI switching effect, the paramagnetic Fe atoms that remain in the undissociated Au@FePt are excellent negative T2WI contrast agents, while the released Fe^2+^ is a positive T1WI enhancer. As shown in Fig. 2F, the T2-signal-to-background (SBR) in a neutral condition (pH = 7.4) was 14.9, lower T2-SBR values were observed when Au@FePt NPs were exposed to acidic conditions, with values of 13.8, 13.3 and 10.64, determined at pH 6.5, 5.8, and 4.0, respectively.

### In vitro test for Fenton reaction

In terms of the Fenton reaction, H_2_O_2_ is converted into highly toxic ·OH, which is catalyzed by Fe^2+^. The generated ·OH can efficiently disrupt the key functions of a cancer cell and its structures of biomolecules, such as DNA, proteins, and lipids [47]. The methylene blue (MB) as the dye can be decolored by OH, which was utilized to reveal the ·OH generation ability of Au@FePt via a Fenton reaction [36]. Figure 2G showed that the absorbance of MB dramatically declined after treatment with Au@FePt nanoprobes (Fe concentration: 100 µg/mL) and different concentrations of H_2_O_2_ (0, 2, 4, 8, and 10 mM) (100 μg/mL), indicating an H_2_O_2_-dependent ·OH generation. Furthermore, MB absorbance in the Au@FePt solution gradually decreased with increasing Au@FePt concentration (from 0 to 100 μg/mL), indicating a large amount of ·OH generation after the Fenton reaction from Fe^2+^ under acidic conditions (Fig. 2H). Notably, the intensity of the MB fluorescence gradually decreased as the Au@FePt concentration increased from 0 to 100 mg/mL (Fig. 2I). Overall, the H_2_O_2_ concentration-dependent Fenton effect of Au@FePt could further enhance ·OH generation.

### In vitro cytotoxicity assay of the Au@FePt NPs

**Fig. 3:**
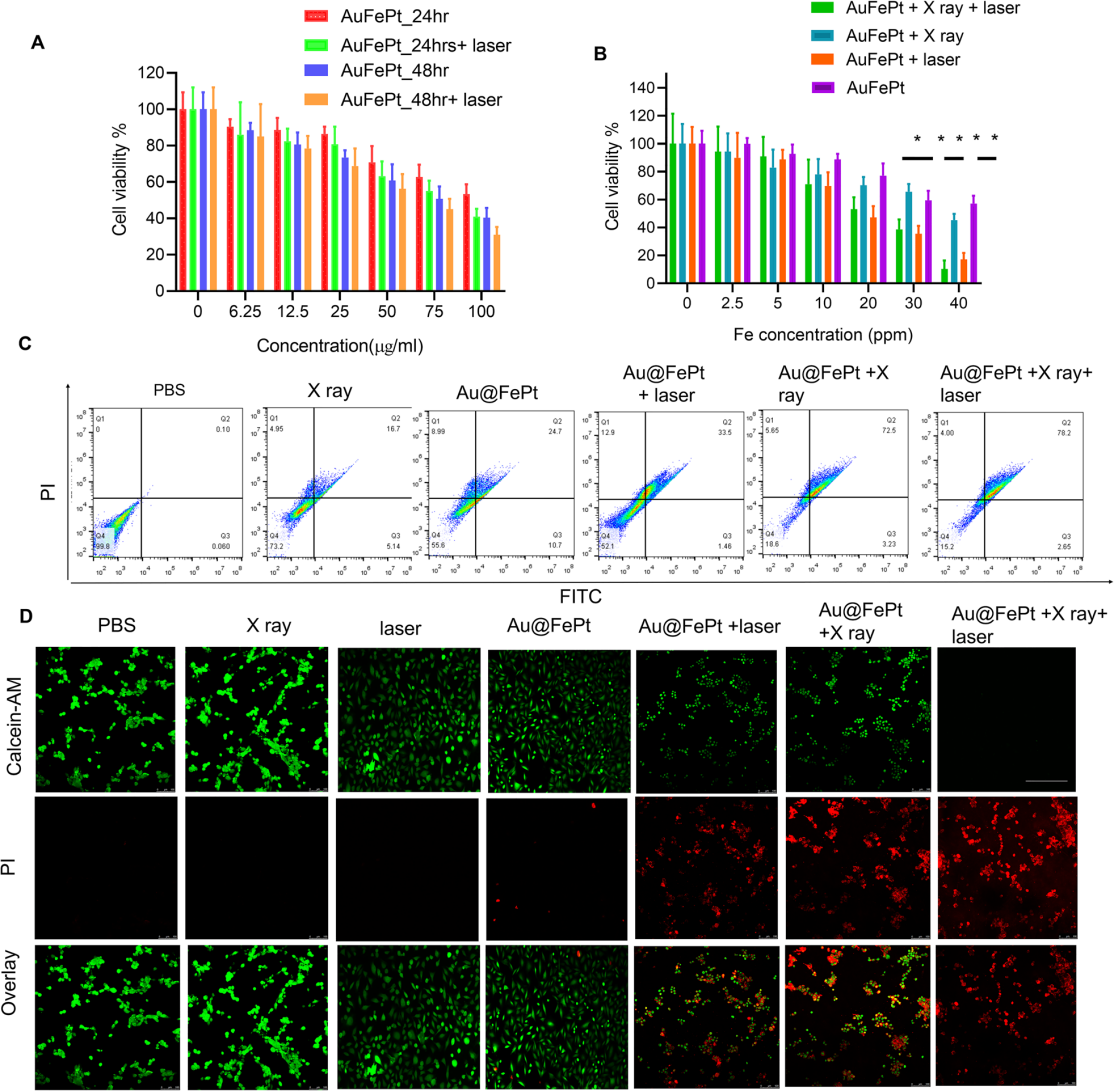
**A** The cell viability of 4T1 cells after treatment with different concentrations of Au@FePt for 24 h and 48 h (n = 5). **B** Cell viability test of Au@FePt and with and without X-ray or laser irradiation for 24 h (n = 5). Data represent mean ± SD from three independent experiments, each performed in five replicates (*P < 0.05, **P < 0.01). **C** Flow cytometry analyses of cell apoptosis by Annexin V-FITC/PI co-staining of different treatment groups. **D** Calcein-AM/PI co-staining of 4T1 cells after the indicated treatments (green: live cells; red: dead cells) of various treated groups (scale bar = 100 μm)

The in vitro cytotoxicity of Au@FePt NPs with photothermal/radiation synergetic therapy against 4T1 cells under different treatment times was assessed by the standard CCK-8 assay. As shown in Fig. 3A, the cytotoxicity result revealed that the Au@FePt + laser (1.2 W/cm^2^, 5 min) group’s cytotoxicity (1.2 W/cm^2^, 5 min) was 1.2 and 2.6-fold higher than that of Au@FePt groups at 24 and 48 h, respectively. As shown in Fig. 3A, the IC_50_ value (i.e., the drug concentrations required to induce 50% cell death within a certain period) of Au@FePt without laser irradiation was 150 μg/mL and 109.7 μg/mL after 24 and 48 h of incubation, respectively. Compared to the IC_50_ of Au@FePt receiving laser irradiation (108.9 μg/mL and 74.5 μg/mL) after 24 h and 48 h incubation, the cell viability of Au@FePt notably diminished, demonstrating that the AuNCs encapsulation of FePt exhibited higher cytotoxicity upon 1064 nm laser irradiation (1.2 W/cm^2^, 5 min). Besides, the cytotoxicity was ranked as follows: Au@FePt + NIR + X-ray > Au@FePt + laser > Au@FePt + X-ray > Au@FePt in Fig. 3B. The data indicated that the Au@FePt without laser and X-ray exposure revealed relatively weaker cytotoxicity on cancer cells. Treatment with Au@FePt under laser and X-ray exposure resulted in higher cytotoxicity towards 4T1 cells than Au@FePt + laser and Au@FePt + X-ray groups, indicating that mild PTT and XDT induced higher cytotoxicity in cancer cells, as shown in Fig. 3C. Flow cytometry was used to analyze the cell death pathways of Au@FePt under various treatments compared to control groups after staining with an annexin V-FITC/PI kit. Au@FePt with laser and X-ray had the strongest CDT ability under mild PTT and could induce early apoptosis accompanied by secondary necrotic/late apoptosis (78.5%), consistent with cell viability assay results (Fig. 3C). The results showed that the apoptotic rates of the PBS group, X ray, Au@FePt, Au@FePt + laser, Au@FePt + X rayand Au@FePt + X-ray groups were ~ 0.1%, ~ 16.7%, ~ 24.7%, ~ 33.5, 72.5% and 78.2%, respectively (Fig. 3D), which were similar to those of the CCK8 assay.

In addition, the treatment effect was further confirmed by the live/dead cell staining assay was conducted using a calcein-AM/PI kit to further verify the synergetic low-temperature PTT with XDT/CDT effect of Au@FePt. Under light irradiation for 5 min (1064 nm, 1.2 W/cm^2^), Au@FePt + laser and X-ray induced approximately 90% cell death, which was consistent with CCK8 results (Fig. 3D). The cell imaging data demonstrated that the entirety of the cell nuclei in the Au@FePt + laser + X-ray group had a strong PI signal related to chromatin condensation, indicating that cell death had happened compared to the X-ray with or without laser irradiation as shown above [48]. Although the killing efficiency of cancer cells via Au@FePt was greatly weakened without X-ray treatment, the PTT and CDT effects of Au@FePt NPs were maintained, further highlighting the important role of Au@FePt NPs in CDT-combined mild PTT. Moreover, without the use of a laser, fluorescence cell imaging revealed that the XDT/CDT consecutively damaged the cell membrane and induced apoptosis in the Au@FePt + X-ray groups.

### Transcriptomic signatures through RNA sequencing by Au@FePt treatment in breast cancer

**Fig. 4:**
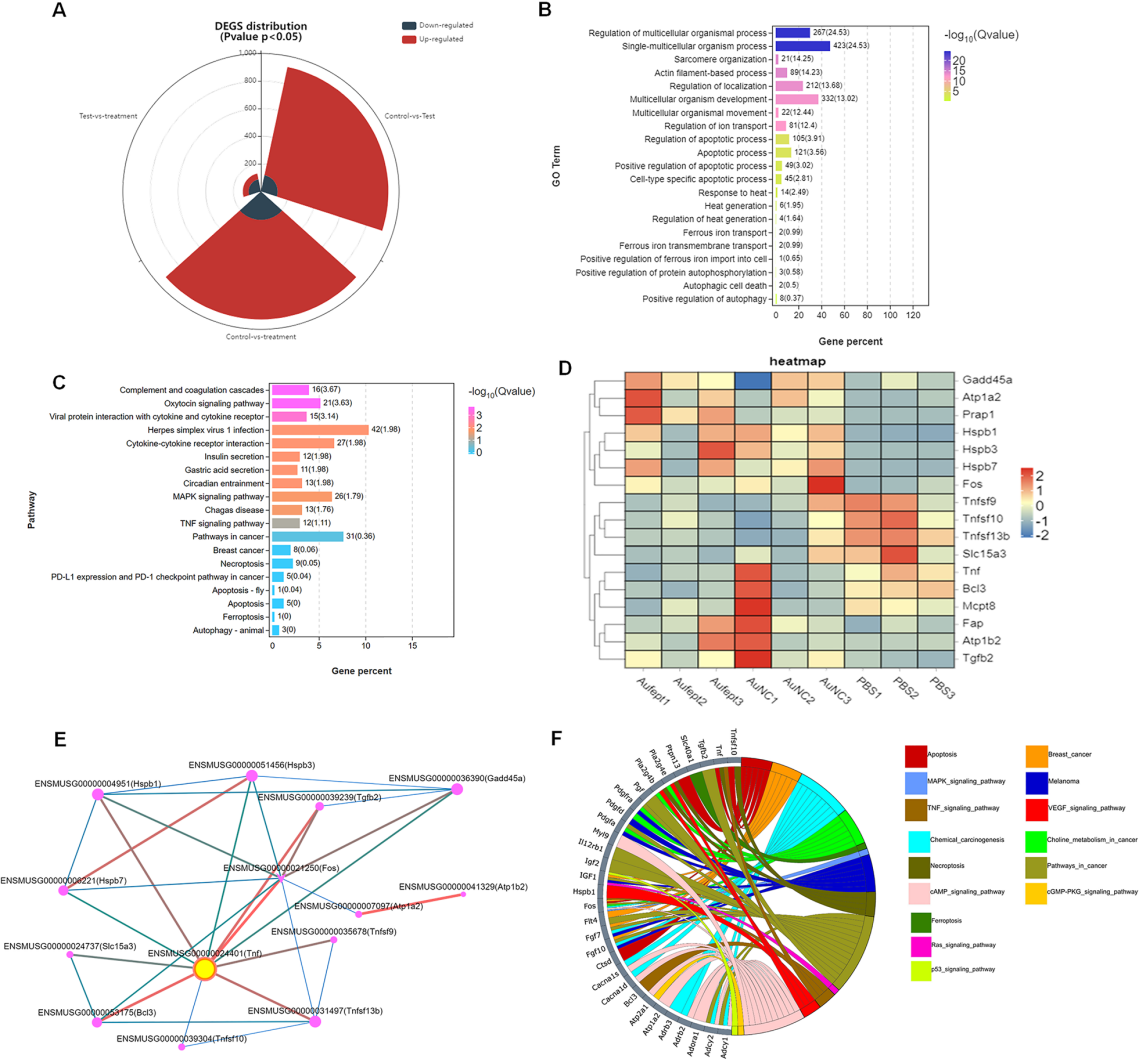
RNA-seq and differentially expressed genes (DEGs) results. RNA sequencing of 143B cells after treatment of normal saline as Control, Ca, DPA, or DPAC. **A** The landscape of up-and-down regulated DEGs number distributed between groups that compare with each other (PBS + laser vs Au@FePt + laser + X-ray, PBS + laser vs AuNCs + laser, AuNCs + laser vs Au@FePt + laser + X-ray). log_2_FC > 1.5 was defined as upregulation (red) and log_2_FC < − 1.5 was defined as downregulation (gray-blue). The landscape of DEGs distribution between control (PBS treatment) and Au@FePt + laser + X-ray groups. Colored points represent FDR < 0.05 (− log (FDR) ≥ 1.5, dashed line), log_2_FC > 1.5 upregulated genes (yellow) and log_2_FC < − 1.5 downregulated genes (red). **B** GO enrichment analysis of DEGs for the Au@FePt + laser + X-ray treated mice. A Fisher’s exact test displayed significance levels (− log_10_ Q-value, size). The number of genes enriched in each GO term and the Q value were marked next to each bar. **C** Bar plot showing the top 19 enriched KEGG pathways for DEGs in the Au@FePt + laser + X-ray treated mice. The number of genes enriched in each pathway and the Q value was marked next to each bar. KEGG pathway analysis of from Au@FePt + laser + X-ray treated mice and PBS treated glioblastoma. enriched p38 mitogen-activated protein kinase (MAPK) signaling pathway and tumor necrosis factor (TNF) signaling pathway. **D** A heatmap shows apoptosis/ferroptosis-related differentially expressed genes between treatments. Color intensity represents the degree of expression value of this gene after standardized treatment in each sample. Relative expression levels are shown in red (up-regulation) and blue (down-regulation). **E** PPI network demonstrating specific gene interaction pathways derived from representative DEGs, and yellow nodes represented the locations of a hub node. PPI network consists of specific genes encoded by representative DEGs. **F** The Circos plot visualizes significant associations between 32 representative DEGs and KEGG pathways in a different color. Icons marked the 15 cancer-relative KEGG pathways enriched by representative DEGs

To evaluate the transcription profile in Au@FePt treated, the RNA sequencing analysis of the 4T1 tumor model with different treatments was performed. The distribution of differentially expressed genes (DEGs) amount between different groups was shown as a stacked-rose chart (Fig. 4A). Given that the Au@FePt synergetic treatment resulted in the greatest change in DEG number when compared to other treated groups, we investigated the potential functional and mechanism pathways enriched in the Au@FePt + laser + X-ray group’s DEGs. The gene ontology (GO) enrichment analysis showed that the DEGs expression of Au@FePt + laser + X-ray treatment was typically enriched in the biological processes, involving “Response to heat”, “Heat generation”, “Apoptosis process”, “Regulation of iron transport, and “Positive regulation of apoptosis” (Fig. 4B). Furthermore, the Kyoto Encyclopedia of Genes and Genomes (KEGG) pathway enrichment analysis revealed several pathways related to cancer pathology that were regulated by the AuFeT + laser + X-ray treatment (Fig. 4C). Furthermore, a heatmap was plotted to depict the distinct expression patterns of 18 representative DEGs among different treated groups (Fig. 4D). Up- and down-regulated genes in each group compared with those of the PBS were set to GO enrichment analysis. To further characterize the relational links of these representative genes, a protein–protein interaction (PPI) network indicated the relational associations among encoded genes. The size and color of the rings represent the importance of different genes in the PPI network. Results in PPI indicate the hub gene was TNF, which encodes the tumor necrosis factor-alpha that has been a critical factor in tackling breast cancer (Fig. 4E). In-depth, to visualize relations between genes and pathways, we provide a Circos plot to depict the subordination of 32 representative DEGs. The plot shows that the “cAMP signaling pathway” and “cancer pathway” have the highest number of genes enriched. Additionally, other pathways, including “MAPK signaling pathway,” “TNF signaling pathway,” “Apoptotic pathway,” “Pathway in cell death,” “Ferroptosis pathway,” “Ras signaling pathway,” and “P53 signaling pathway” also participated in the gene expression change of Au@FePt + laser + X-ray treatment (Fig. 4F). To be brief, an extensive number of DEGs were identified after Au@FePt + laser + X-ray treatment, and Au@FePt + laser + X-ray could cause transcriptional dysregulation and affect oncogenic signaling in 4T1 tumor cells, especially in apoptosis and ferroptosis. Bioinformatic results revealed that biomineralization could tremendously inhibit cancer progression by disrupting the heat generation process and inducing cell cycle arrest, apoptosis, and ferroptosis.

### Intracellular ROS generation

**Fig. 5:**
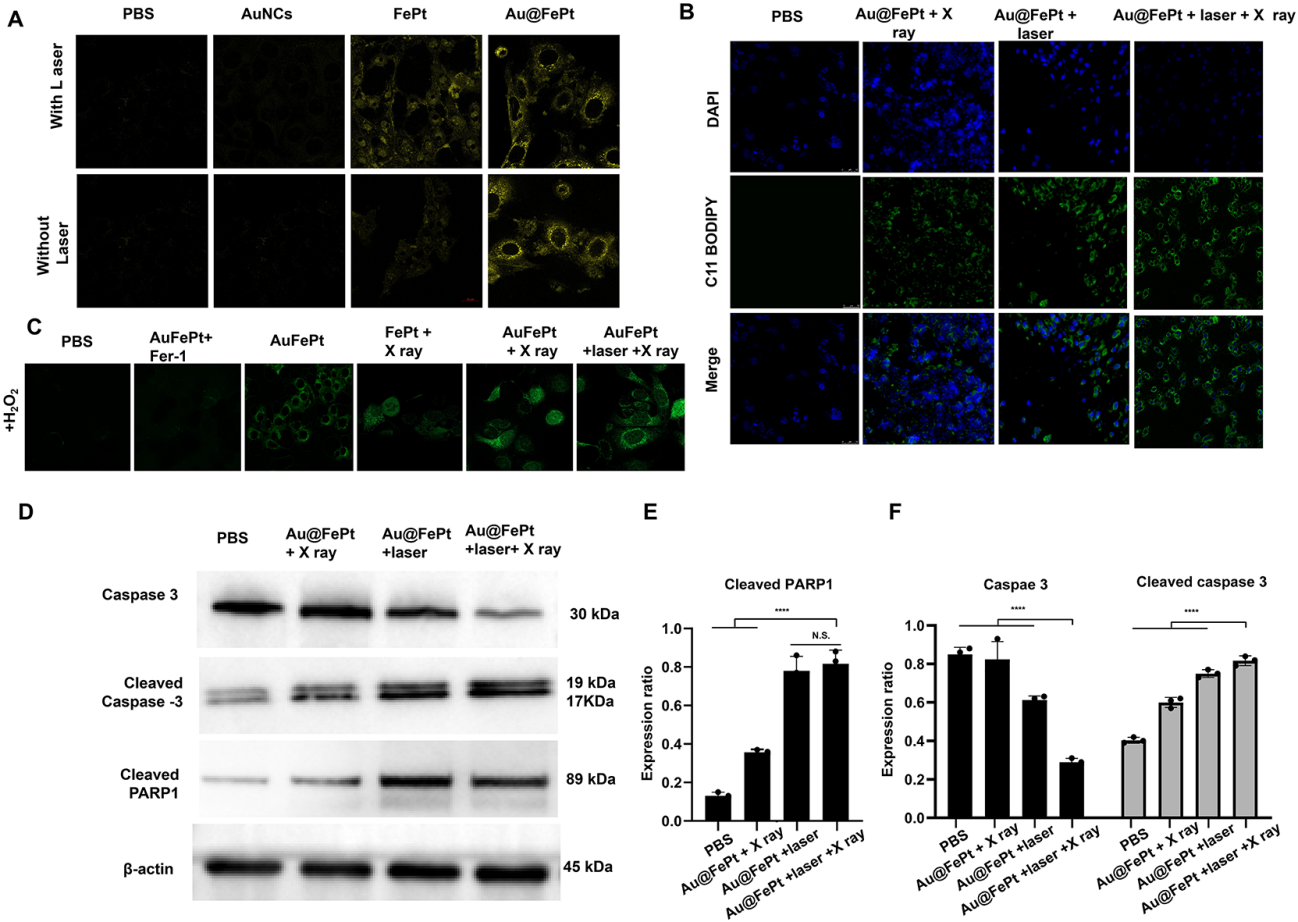
**A** Fe^2+^ fluorescence staining without and with FePt nanoprobes treatment in 4T1 cells (scale bar: 50 μm). **B** Fluorescence images of lipid peroxide in 4T1 cells after treatment with PBS, FePt-Fer-1, Au@FePt + NIR, and Au@FePt + NIR + X-ray (scale bar: 50 μm). **C** Intracellular ROS generation was detected by the DCFH-DA probe after various treatments in 4T1 cells (Fe concentration: 5 mM, X-ray: 4 Gy) (scale bar: 50 μm) (*P < 0.05, **P < 0.01). **D** Western blot analysis of apoptosis-associated proteins after 48 h treatment within different nanoprobe-incubated cells. **E**, **F** Quantitative measurement of apoptosis-associated proteins in different nanoprobe-incubated cells during western blot imaging process (n = 3)

To reveal the in vitro therapeutic efficacy, the promising advantage of AuNCs loaded FePt was attributed to cell internalization at the tumor microenvironment for precisely enhanced apoptosis/ferroptosis effects in breast cancer. The generation of Fe^2+^ was verified using the Ferro Orange fluorescence staining assay, whose fluorescence intensity increased as the Fe^2+^ was enriched in the cells. Au@FePt NPs treated 4T1 cancer cells (+NIR-II laser irradiation) demonstrated an enhanced fluorescence signal than the Au@FePt and PBS group (Fig. 5A), indicating that co-incubation with laser enhanced the Fe^2+^concentration in 4T1 cells. Besides, the BODIPY581/591-C11 probe was added to detect the LPO level in the 4T1 cell. Fer-1 (a ferroptosis inhibitor) was added to 4T1 cells prior to the BODIPY581/591-C11 assay to directly investigate the LPO effect on Au@FePt + laser + X-ray-induced cell death. In Fig. 5B, the Au@FePt with laser plus X-ray radiation group revealed the strongest green fluorescence, followed by the Au@FePt + laser, Au@FePt + X ray, and PBS groups as shown in Fig. 5B. The cell images result demonstrated that the Au@FePt mild PTT plus X-ray radiation group produces a great amount of LPO, which was combined with the ROS production and GPX4 depletion result [35].

To investigate the enhanced ROS generation ability of the Au@FePt nanoprobe, the change in oxidative stress was investigated using DCFH-DA as a ROS sensor probe, which can be oxidized by ROS into 2′,7′-dichlorofuorescein (DCF), a green fluorescence-emitting molecule. As illustrated in Fig. 5C, to mimic the tumor microenvironment overexpressing H_2_O_2_, 4T1 cells were incubated with H_2_O_2_ while undergoing different treatments. Compared with the PBS group, Au@FePt-treated 4T1 cells exhibited a certain amount of green fluorescence, indicating that ROS generation was detected, revealing that Fe^2+^ released from Au@FePt undergoes a Fenton reaction in the presence of H_2_O_2_ to play a role in CDT. However, in the presence of Fer-1, Au@FePt-treated cells exhibited extremely low fluorescence signals, indicating that Fer-1 inhibited the function of Fe^2+^and further illustrating that it is the Fe^2+^ in Au@FePt that plays a role in ROS generation. Au@FePt-treated cells showed the highest fluorescence signal after exposure to mild PTT and X-ray radiation, indicating the most ROS generation, which illustrated the excellent combined CDT/PTT/XRT therapeutic effect of the nanoprobe. Ferroptosis acts as one of the typical RCD based on Fe-dependent oxidative damage and is induced by the reduction of glutathione peroxidase 4 (GPX4). Down-regulation of GPX4 would result in the formation of lipid peroxide (LPO) in cancer cells.

### Activated apoptosis and ferroptosis through syngenetic therapy

To examine the cell death mechanism, a western blot was employed to monitor the cell death pathway upon syngenetic therapy, which indicates the cancer cell pathway via quantitative in situ activation of intracellular apoptosis/ferroptosis-related proteins. Overall, the above results strongly confirmed that Au@FePt + laser + X-ray treated cells could inhibit autophagy in cancer cells, thus sensitizing the Au@FePt-mediated PTT and CDT-mediated syngenetic therapeutic effect.

The RCD mechanism via various protein markers of apoptosis was further assessed by western blot, including caspase-3, cleaved caspase 3 and cleaved poly (ADP-ribose) polymerase (PARP) [49]. The western blot results revealed that Au@FePt-induced XDT/CDT/PTT upon laser irradiation treatment up-regulated the expression of caspase-3 (Fig. 5D). To reveal the detailed cell death mechanism of different treatments during the bidirectional crosstalk between apoptosis and ferroptosis, we study the apoptosis behavior with NIR-II laser irradiation or with X-ray in various groups. Apoptosis was assessed by caspase 3, cleaved caspase-3, and cleaved poly (ADP-ribose) polymerase (PARP). As shown in Fig. 5E, F, the Au@FePt receiving laser with X-ray exhibited a higher apoptosis rate than other groups, such as Au@FePt + laser and Au@FePt + X-ray and PBS control group. Subsequently, PARP-1 was cleaved to represent a signal of apoptosis as shown in Fig. 5F. Those results demonstrated that both intrinsic and extrinsic apoptotic pathways were activated by the synergistic photothermal and enhanced chemotherapy therapies [42, 43, 50].

### In vivo MRI and PA imaging

**Fig. 6:**
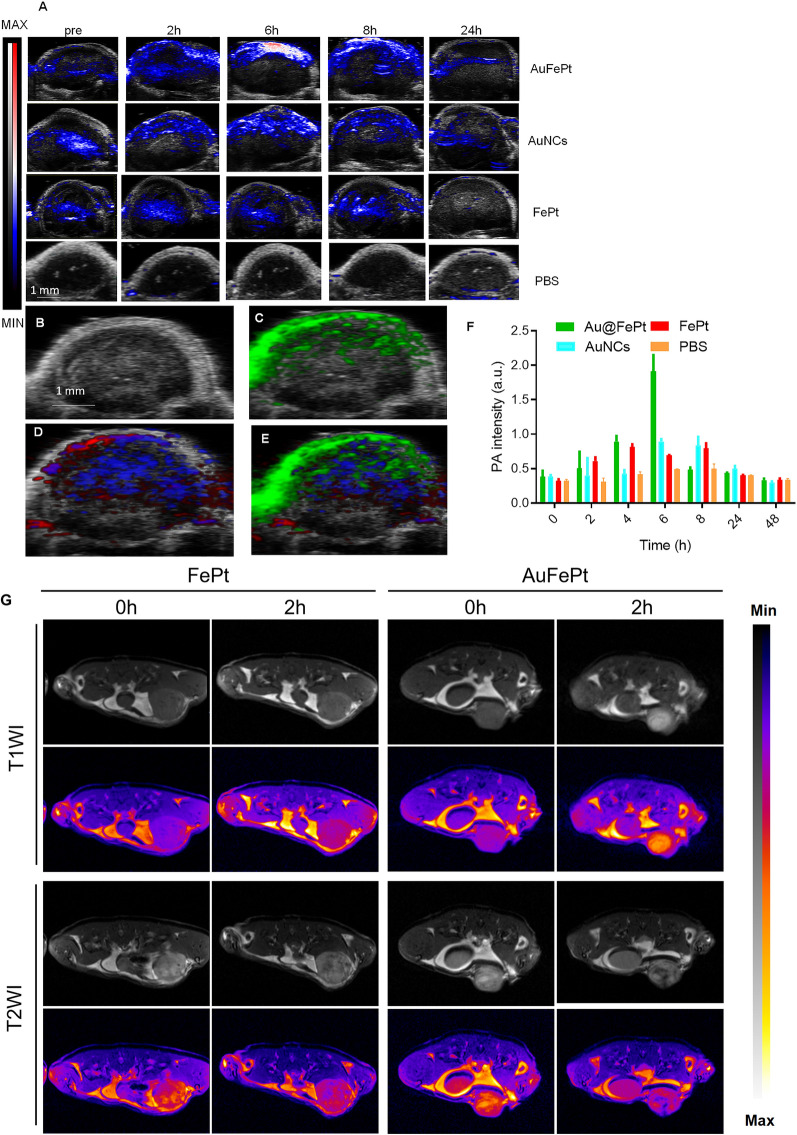
Monitoring different nanoprobe accumulation with photoacoustic imaging. **A** Heat map of NP accumulation in tumors in PA-US overlay images respectively with different nanoprobe into mice bearing 4T1 tumors after 0 h, 2 h, 6 h, 8 h and 24 h (n = 3). **B** In vivo ultrasound image of a subcutaneous implanted 4T1 tumor. **C** Heat map of PA signals from targeted nanoprobe injected in mice implanted with 4T1 tumor (green). **D** Heat map of oxygenated hemoglobin (red) and deoxygenated hemoglobin (blue) of the same 4T1 tumor. **E** Overlaid with ultrasound image and targeted nanoprobe, oxygenated hemoglobin, and deoxygenated hemoglobin PA signals. **F** Average PA intensity increment at 850 nm as a function post-injection time with different nanoprobe into mice bearing 4T1 tumors after 0 h, 2 h, 4 h, 6 h, 8 h, 24 h, and 48 h (n = 3). Error bars represent standard deviation. *p < 0.05. **G** In vivo T1WI and T2WI of 4T1 tumors at various time points after intravenous injection of Au@FePt nanoprobe and FePt nanoprobe

To reveal the in vivo targeting ability of Au@FePt, the FePt and AuNCs NPs (10 mg/kg) were administrated via tail vein into orthotopic 4T1 tumor-bearing mice. Vevo 2100 photoacoustic system was used to examine the photoacoustic image and signal changes. These PA overlay ultrasound images demonstrated a local distribution of the Au@FePt over 24 h. The PA images revealed that the tumor was lightened after injection of Au@FePt nanoprobes at 6 h post-injection which then started to decline and returned to the pre-injection level at the end, whereas the PBS control group exhibited minor PA signal change during the entire period. The PA signal in the breast cancer site of Au@FePt-treated mice elevated over time and reached its highest level after 6 h post-injection, which achieved a 2.8-fold PA signal enhancement over AuNCs and FePt at 6 h (Fig. 6A). The increased local concentration of Au@FePt in a tumor at this time point revealed the association between the endothelial cells based on α_v_β_3_ integrin which is highly expressed in the anagenesis of the vascular structure resulting in the nanoparticle targeting the tumor site directly. The creation of a leaky vasculature, which was characterized as increased blood flow around the tumor, may have contributed to the hypoxic core that was seen.The signal from targeted Au@FePt can be deconvoluted via PA multispectral scanning with specific absorption spectra for HbO_2_ and Hb in the tumor region (Fig. 6B–E) [51]. Following intravenous delivery, the photoacoustic intensities increased by a factor of 2.1, revealing the potential of Au@FePt NPs as capable photoacoustic contrast agents for tumor imaging (Fig. 6F).

Dual imaging with PAI combined with MRI exhibited superiority both in morphological and functional imaging. Furthermore, the performance of Au@FePt NPs as contrast agents for dual-mode in vivo tumor imaging was investigated. Herein, the images of T1-weighted and T2-weighted 4T1 tumor-bearing mice were recorded, and the corresponding signal at different time points was measured. As shown in Fig. 6G, previous ex vivo MRI switching data revealed that during Au@FePt corrosion, the relaxation rate changed from T2 to T1 in an acidic condition. Therefore, the identical sections of breast tumor-bearing animals were imaged using T1 and T2 weighting, and the signal-to-background ratios (SBRs) at various time intervals were computed. After intravenous injection of Au@FePt nanoprobes, the tumors only showed an increased T1WI signal at tumor locations, indicating Fe^2+^ release at the tumor sites. Besides, tumor areas showed negative T2WI enhancement, which is thought to be a sign that Au@FePt have been retained in these tumors. After calculating the ratios of T1 SBR and T2 SBR, it was discovered that the ratio significantly increased for two hours after injection (Additional file 1: Fig. S7A–C). Besides, for the FePt (2 mg/mL for Fept; 200 µL) as non-targeted group was administered to the mice bearing 4T1 through the tail vein and imaged continuously for 2 days, every 2 h per image. The results indicated that targeted (Au@FePt) show high uptake in the tumor site after tail vein injection compared to the FePt group (Additional file 1: Fig. S7D–F). Overall, the Au@FePt can enhance positive T1/negative T2 contrast in MRI and combine with the new imaging technology of photoacoustic imaging to provide a high tumor imaging contrast to ensure sufficient imaging sensitivity. Furthermore, PEG-modified phospholipid bilayer shell release of Au@FePt in a pH-sensitive manner provides in situ tumor targeting and achieves the mild photothermal/radiation synergetic therapy effect of potentiating ferroptosis effect. Compared with traditional methods, multifunctional nanoparticles have advantages that can realize dual-modal new imaging and multi-process synergistic therapy.

### In vivo therapeutic efficacy of Au@FePt through combined synergetic therapy

**Fig. 7:**
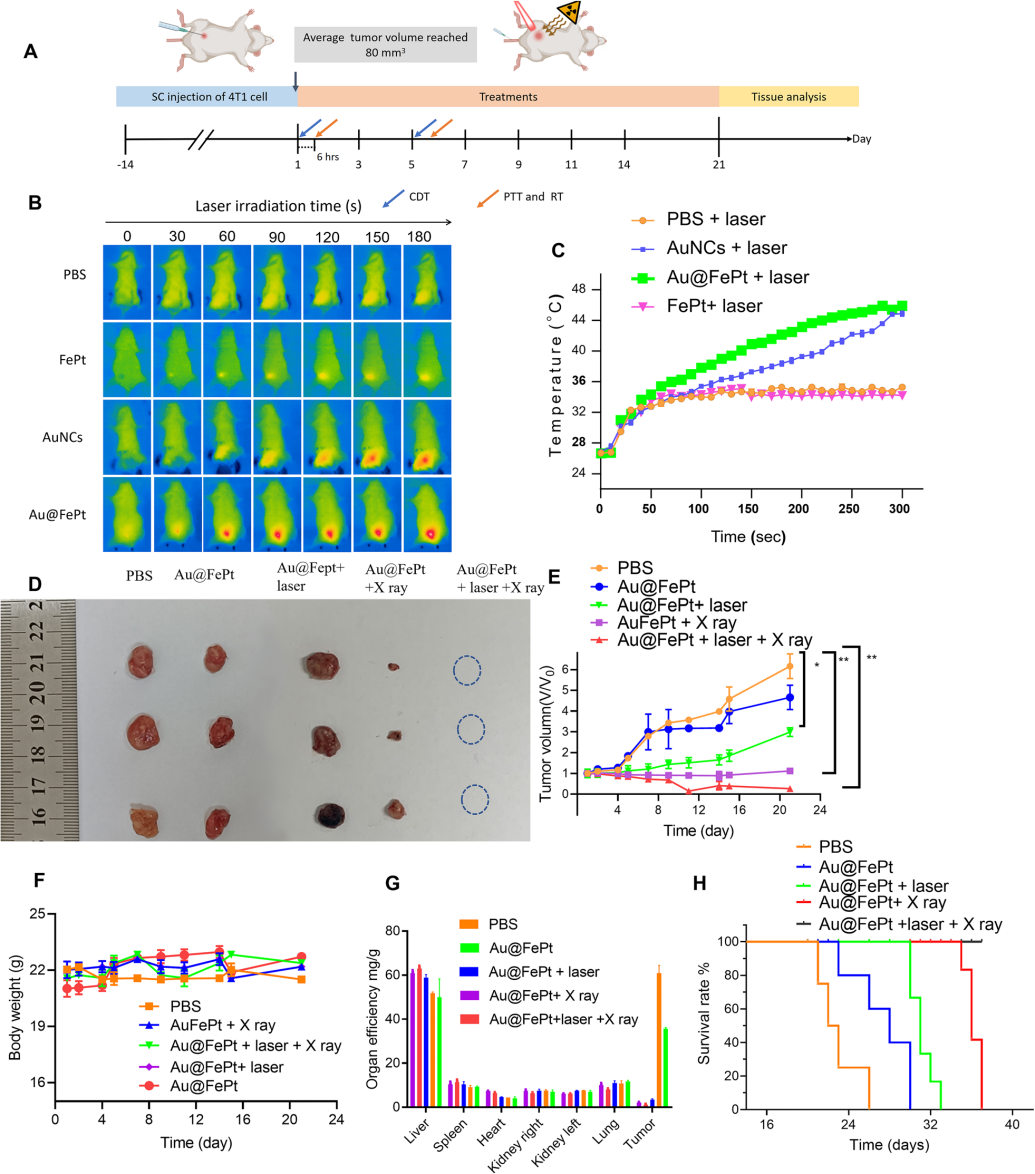
In vivo XDT/CDT/PTT therapy of the 4T1 tumor-bearing mice after injection of PBS, Au@FePt with and without laser irradiation or X-ray after 6 h of injection (5 mg/kg, 1.2 W/cm^2^). **A** Schedule of therapeutic and assessment. **B** Thermal images and **C** corresponding temperature changes of tumor site of PBS, Au@FePt with and without laser irradiation after 6 h post-injection (n = 4). **D** Photographs of the tumor systematically administrated after PBS, Au@FePt and Au@Fept + X ray, Au@FePt + laser (1.2 W/cm^2^) and Au@FePt + laser + X-ray (n = 4). **E** Relative tumor-growth curve. Insert the photographs of relevant tumors originating from each group in **D**. **F** Body weight after various treatments indicated in 21 days (n = 4). **G**, **H** Survival curve and organ efficiency of tumor-bearing mice treated by various groups during 42 days (n = 4)

To reveal the anti-tumor efficacy for different treatment groups, the in vivo treatment strategy of the orthotopic breast tumor model was observed in Fig. 7A. For synergistic treatment groups as shown in Fig. 7B, the mice were also injected with Au@FePt (dose: 10 mg/kg) and then irradiated with a NIR laser at 1064 nm with 1.2 W/cm^2^ for 5 min at 6 h post-injection, and the IR thermal camera recording for the breast cancer site showed a dramatically increasing temperature over time. The temperature quickly rose to 44.5 °C and then remained constant at 45.8 °C for 5 min after 1064 nm laser exposure for the Au@FePt treated group (Fig. 7B). Subsequently, mice were injected with AuNCs (10 mg/kg based on the weight of Au concentration) and FePt (5 mg/kg based on the weight of Fe concentration) and irradiated with laser light 6 h after the injection, respectively. The AuNCs treated group also strengthened the PTT effect whereas the FePt treated group displayed a moderate temperature arise to 32 °C. Nevertheless, after 5 min of laser irradiation, the PBS control group barely changed temperature (from 26 to 32 °C) at the tumor site as shown in (Fig. 7C). The change in the tumor volume curve and each tumor photo for each group are illustrated in Fig. 7D, E and Additional file 1: Fig. S5, respectively. The significant tumor accumulation of Au@FePt + laser demonstrated superior anti-tumor efficacy, which was attributed to the AuNCs delivery system’s extraordinary heat-generating ability. As expected, treatment with the Au@FePt NPs + X-ray + laser resulted in an excellent therapeutic effect in inhibiting tumor growth, demonstrating the XDT/CDT/PTT combination therapy effect. In comparison, the 4T1 tumors were injected with an equal volume of PBS dispersion, and the size of the tumor growth was faster and uncontrolled.

Also, there was no significant body weight variation after injection of PBS, Au@FePt, Au@FePt + laser, Au@FePt + X-ray and Au@FePt + laser + X-ray under laser irradiation and X-ray treatment as illustrated in Fig. 7F. Figure 7G also revealed the change in organ efficiency of each organ in the different treatment groups over 21 days. This result demonstrated significant tumor inhibition in 4T1 tumor-bearing mice injected with Au@FePt NPs with synergistic treatment. Kaplan–Meier survival curves were used to assess therapeutic efficacy. The survival curve was measured every day for up to 3 weeks as indicated in Fig. 7H. When compared to Au@FePt NPs + X-ray or Au@FePt + laser groups, in which cancer grew at a moderate speed due to the single effect of mild PTT with XDT/CDT/PTT in cancer cells to inhibit tumor angiogenesis, the higher survival rate of the Au@FePt + laser + X-ray treated group could be attributed to synergistic therapies that induce inflammation with DNA damage in RCD, thereby downregulating caspase expression in the hypoxic tumor microenvironment via dual induction of ferroptosis and apoptosis [27].

**Fig. 8:**
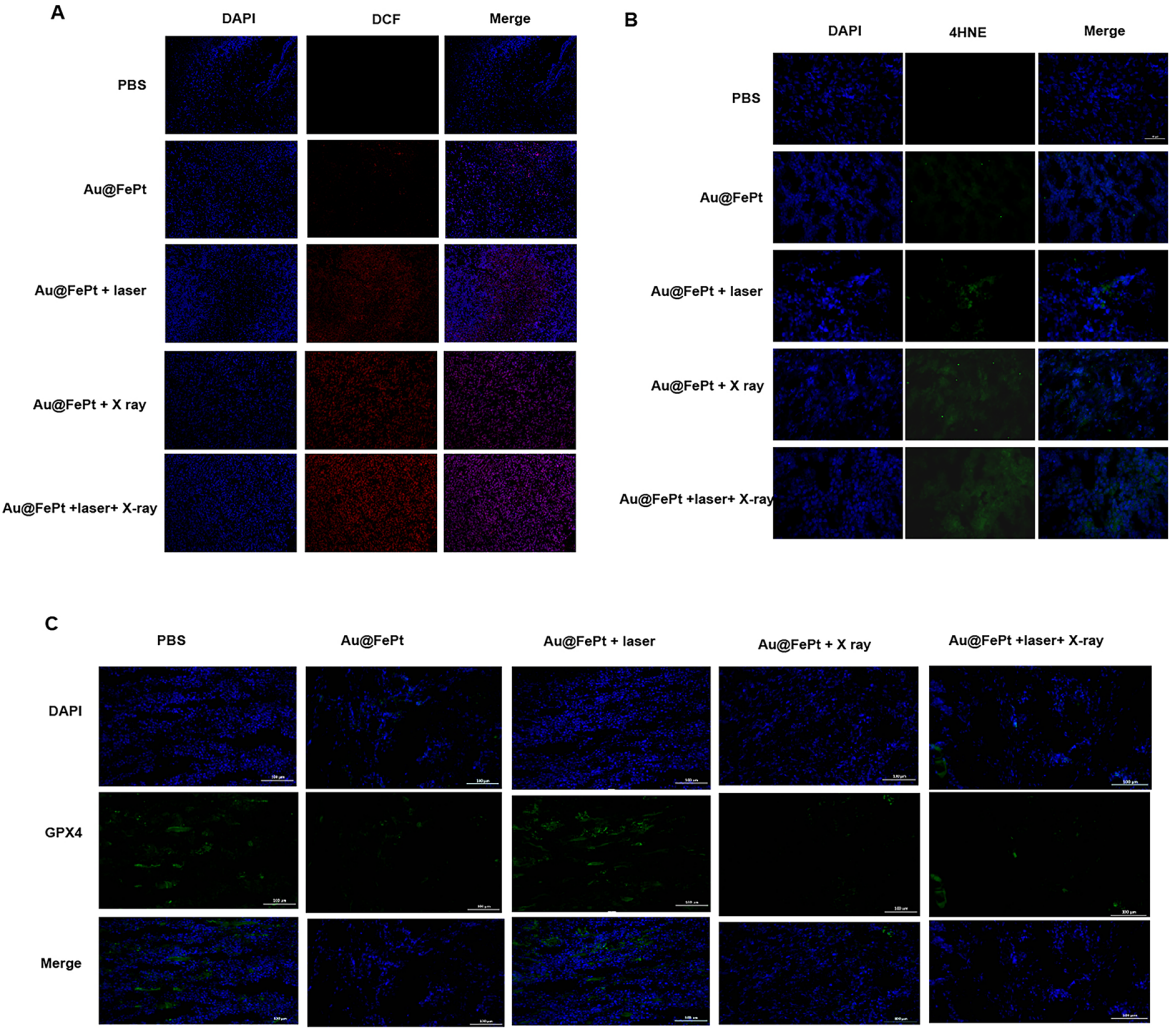
Intratumoral production of **A** ROS and **B** 4-HNE obtained from tumor tissue slices of mice receiving different treatments group. Mice were sacrificed and tumors were dissected at 24 h post-irradiation. Scale bar: 50 µm for immunofluorescence images and 50 µm for H&E staining images. **C** Intratumoral production of GPX4 activity measurement. Data represent the mean (± standard deviation, SD) of 3 independent experiments

To further reveal ferroptosis phenomena, an immunohistochemical examination of tumor sections taken from the treated animals was performed. As shown in Fig. 8A, both Au@FePt-treated groups exposed to an NIR laser and X ray irradiation had higher ROS levels. A greater degree of oxidative stress was observed in the mice that were administered Au@FePt + laser + X-ray than in those that were administered Au@FePt + X ray and Au@FePt + laser. Furthermore, as shown in Fig. 8B, the mice treated with Au@FePt + NIR + X-ray produced intratumoral 4-hydroxynonenal (4-HNE), a significant class of reactive lipid species, indicating that lipid peroxidation contributes to tumor cell death [52]. Previous research indicated that under oxidative stress, such as radiation, the excess of free radicals leads to lipid peroxidation which confirmed the involvement of 4-HNE in tumor cell death [53].

These pathological changes were accompanied by decreased GPX4 activity, which links ferroptosis to the function of key tumor suppressor pathways (Fig. 8C). Collectively, Au@FePt + laser + X-ray resulted in sufficient intratumoral GPX4 inactivation as well as subsequent lipid peroxidation, leading to complete tumor eradication. Thus, the use of Au@FePt + laser + X-ray is a promising therapeutic strategy for the ablation of breast cancer. A certain portion of tumor cell death via the ferroptosis/apoptosis pathway was observed for groups of Au@FePt + laser + X-ray, whereas control groups (PBS + laser) revealed no adverse effect on the tumor cell damage (Additional file 1: Fig. S6). Moreover, an IHC assay with CD31, HIF-1α, and PNAC staining and an immunofluorescent study of tumor tissues were performed (Additional file 1: Fig. S8). Apparent shrinkage of cell morphology and breakdown of tumor tissue were noticed in the Au@FePt + laser + X-ray group compared with the PBS + laser group, indicating an effective tumor suppression effect. According to IHC assays with CD31 and PCNA staining, tumor angiogenesis and cancer cell proliferation were significantly reduced in the Au@FePt + laser + X-ray group. Furthermore, the Au@FePt + laser + X-ray group had a high percentage of TUNEL nuclei, implying that apoptosis and ferroptosis may initiate the primary pathways of combined mild PTT with CDT and XDT, ultimately leading to cell death. PBS as a control group revealed the apparent formation of new blood vessels. TUNEL staining of Au@FePt + laser + X-ray treatment reveals t more apoptotic cells than the Au@FePt + laser group and Au@FePt + X ray. The prominent PTT improved therapeutic outcome with the Au@FePt + laser + X-ray in combination with laser irradiation and X-ray radiation, which holds great promise as an effective anti-tumor agent for in vivo tumor therapy.

### Biosafety test and blood analysis

The biosafety test was assessed via H&E staining assay for all nanoprobes in the treated group, and the result revealed no obvious acute toxicity (Additional file 1: Fig. S9). We also examined the biosafety test via blood analysis. The blood chemical indices including alanine aminotransferase (ALT), aspartate aminotransferase (AST), blood urea nitrogen (BUN), plasma uric acid (UA), blood glucose (GLU), and low-density lipoprotein cholesterol (LDL-C) levels were measured and revealed no obvious acute toxicity (Additional file 1: Fig. S10).

## Conclusion

This approach combining dual PAI and MRI-guided synergetic Au-FePt ternary metallic for orthotopic breast cancer in the NIR-II window overcomes specific resistance to apoptosis, which elicits alternative routes such as ferroptosis. The simple Au@FePt with targeted iRGD peptides can enhance mild PTT and CDT with enhanced radiotherapy against the orthotopic tumor. Notably, enhanced mild PTT and CDT/XDT were two major advanced strategies that promote ROS generation through two cascade steps exploiting the tumor micro-environment when the composite nanomedicine Au@FePt enters the tumor. Moreover, RNA sequencing results revealed the up-regulation of typical pathways, such as “the MAPK signaling pathway,” “TNF signaling pathway,” “the apoptotic pathway,” “the ferroptosis pathway,” and “the P53 signaling pathway,” indicating the coordination mechanisms of apoptosis and ferroptosis. Elevation of ROS level and promotion of cell death through mild PTT combined with CDT and via the enhancement of radiotherapy holds the future for nanotherapeutics promoting anti-cancer effects. In addition, our novel Au@FePt design incorporates FePt with MRI contrast properties, which can be combined with PA imaging of AuNCs loaded FePt to create an “all-in-one” platform for imaging-guided precise therapy. This intelligent strategy proposes a future direction for imaging-guided therapy and combined enhancing apoptosis and ferroptosis, which is critical for significant advances in apoptosis resistance for orthotopic breast cancer of synergetic treatment. Therefore, the synergistic therapy via Au@FePt NPs reveals apoptosis and ferroptosis as major cell death pathways for precise PA/MR imaging-guided laser irradiation-activated PTT/XDT/CDT/PTT, thereby overcoming apoptosis resistance [54].

## Supplementary Information

Additional file 1. Additional documents.

## Abbreviations

PTT: Photothermal therapy
ROS: Reactive oxygen species
CDT: Chemodynamic therapy
XDT: X-ray-induced dynamic therapy
PCD: Programmed cell death
MRI: Magnetic resonance imaging
PA: Photoacoustic imaging
Au@FePt NPs: IRGD-PEG/AuNCs@FePt NPs ternary metallic nanoparticles
EDX: Energy-dispersive X-ray spectroscopy
DMEM: Dulbecco’s Modified Eagle Medium
CCK-8: Cell counting kit-8
ALT: Glutamic-pyruvic transaminase
AST: Glutamic oxaloacetic transaminase
ALP: Alkaline phosphatase
BUN: Blood urea nitrogen
UA: Plasma uric acid
GLU: Blood glucose
LDL-C: Low-density lipoprotein cholesterol
