# Correction to: Mild photothermal/radiation therapy potentiates ferroptosis efect for ablation of breast cancer via MRI/PA imaging guided all-in-one strategy

**DOI:** 10.1186/s12951-025-03982-y

**Published:** 2026-03-04

**Authors:** Zhe Zhang, Hsuan Lo, Xingyang Zhao, Wenya Li, Ke Wu, Fanchu Zeng, Shiying Li, Hongzan Sun

**Affiliations:** 1 Department of Radiology, Shengjing Hospital of China Medical University, Sanhao Street No. 36, Heping District, Shenyang, 110004 China; 2 Guangdong Cardiovascular Institute, Guangdong Provincial People’s Hospital, Guangdong Academy of Medical Sciences, Guangzhou, 510080 China; 3 Medical Research InstituteGuangdong Provincial People’s Hospital (Guangdong Academy of Medical Sciences), Southern Medical University, Guangzhou, 510080 China

**Correction: Journal of Nanobiotechnology (2023) 21: 150**

10.1186/s12951-023-01910-6

In this article Fig. 5 appeared incorrectly and have now been corrected in the original publication. For completeness and transparency, the correct and incorrect versions are displayed below. In Fig. 5B, the immunofluorescence images for the Au@FePt + X-ray and Au@FePt + laser groups were inadvertently replaced with images from other groups. Figures 5D-F contain an error in the presented Western blot (WB) data.

In Fig. 8A, the magnification size of the images for the PBS group, Au@FePt group, and Au@FePt + laser group is inconsistent with that of the other groups. In Fig. 8B, the image originally belonging to the PBS and Au@FePt + laser groups were inadvertently replaced with images from other groups. The experiments pertaining to these groups have been repeated to ensure accuracy.

**Incorrect Figure 5**:

**Fig. 5:**
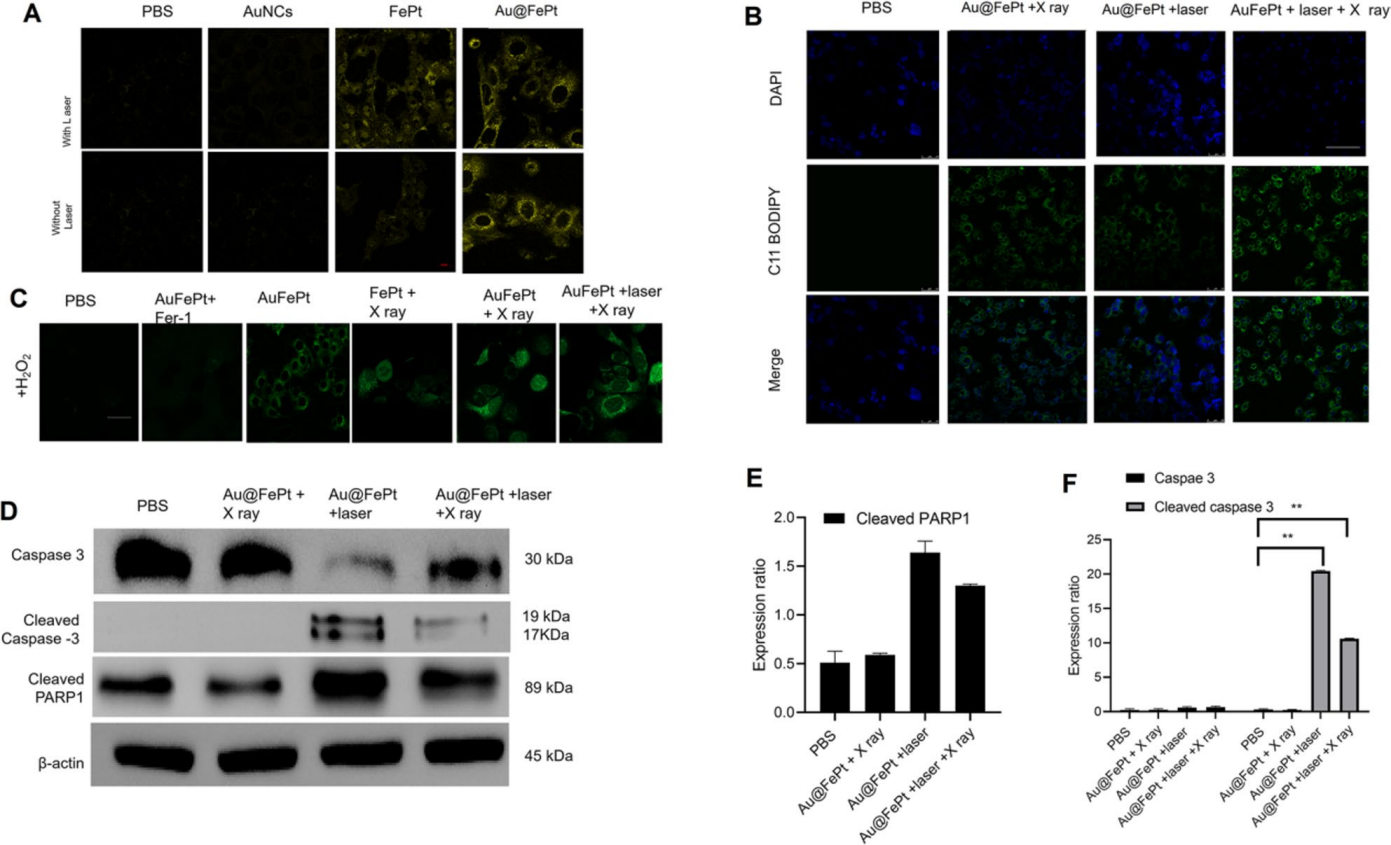
(**A**) Fe2+ fuorescence staining without and with FePt nanoprobes treatment in 4T1 cells (scale bar: 50 μm). (**B**) Fluorescence images of lipid peroxide in 4T1 cells after treatment with PBS, FePt-Fer-1, Au@FePt + NIR, and Au@FePt + NIR + X-ray (scale bar: 50 μm). (**C**) Intracellular ROS generation was detected by the DCFH-DA probe after various treatments in 4T1 cells. (Fe concentration: 5 mM, X-ray: 4 Gy). (scale bar: 50 μm) (*P < 0.05, **P < 0.01). (**D**) Western blot analysis of apoptosis-associated proteins after 48 h treatment within different nanoprobe-incubated cells. (**E** and** F**) Quantitative measurement of apoptosis-associated proteins in different nanoprobe-incubated cells during western blot imaging process (*n* = 3).

**Correct Figure 5**:

**Fig. 5:**
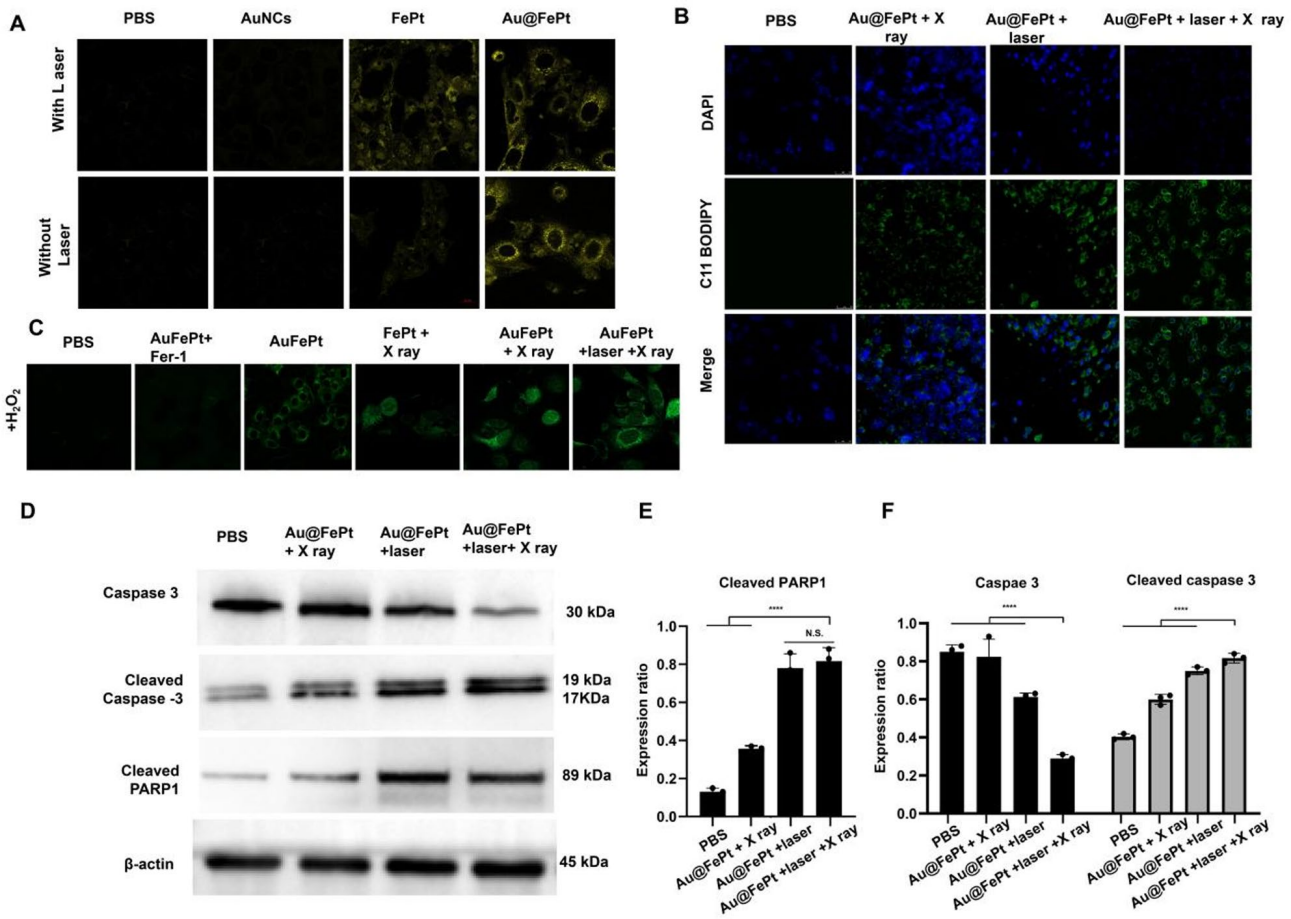
(**A**) Fe^2+^ fuorescence staining without and with FePt nanoprobes treatment in 4T1 cells (scale bar: 50 μm). (**B**) Fluorescence images of lipid peroxide in 4T1 cells after treatment with PBS, FePt-Fer-1, Au@FePt + NIR, and Au@FePt + NIR + X-ray (scale bar: 50 μm). (**C**) Intracellular ROS generation was detected by the DCFH-DA probe after various treatments in 4T1 cells. (Fe concentration: 5 mM, X-ray: 4 Gy). (scale bar: 50 μm) (*P < 0.05, **P < 0.01). (**D**) Western blot analysis of apoptosis-associated proteins after 48 h treatment within different nanoprobe-incubated cells. (**E** and **F**) Quantitative measurement of apoptosis-associated proteins in different nanoprobe-incubated cells during western blot imaging process (*n* = 3)

**Incorrect Figure 8**:

**Fig. 8:**
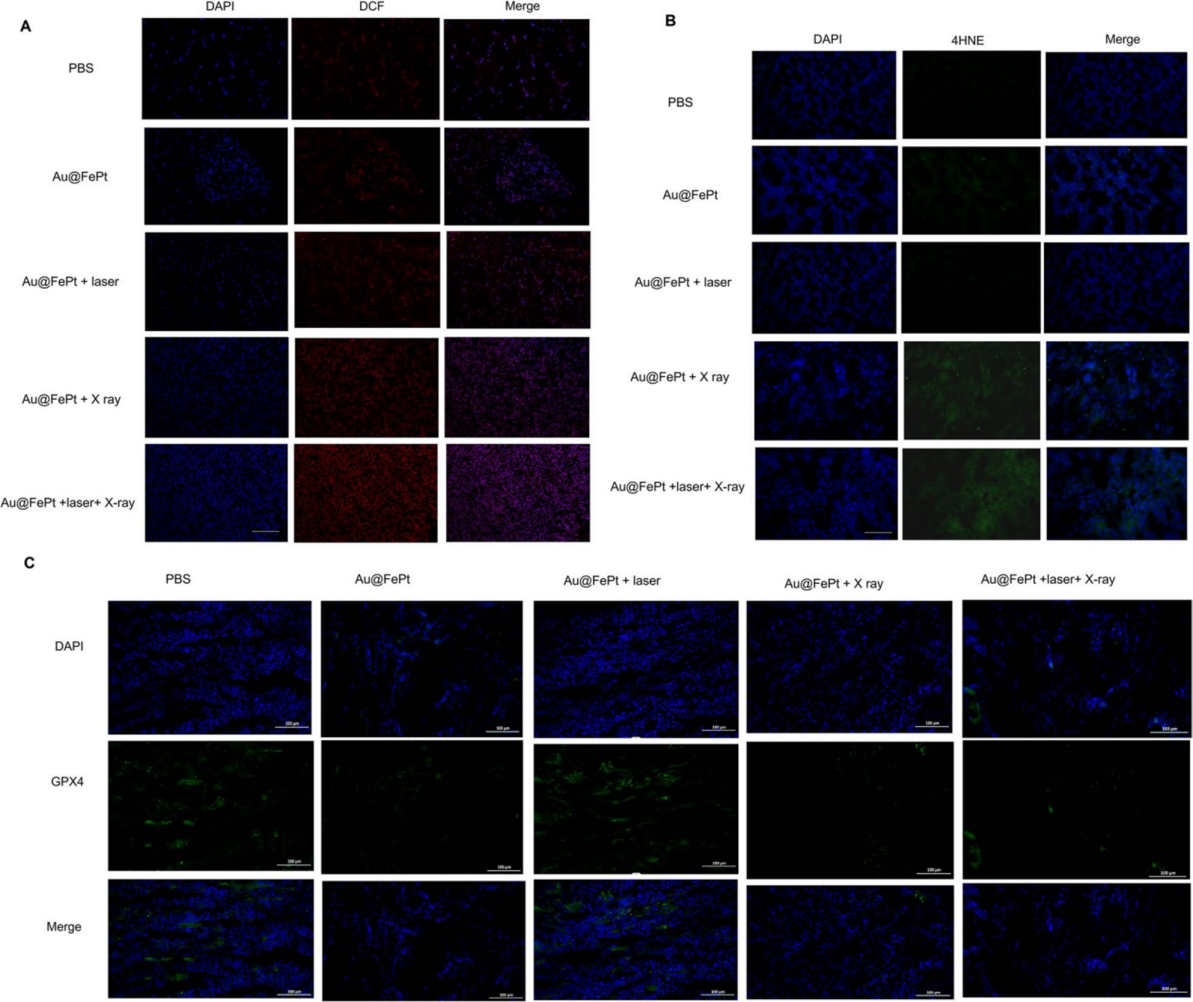
Intratumoral production of (**A**) ROS and (**B**) 4-HNE obtained from tumor tissue slices of mice receiving different treatments group. Mice were sacrificed and tumors were dissected at 24 h post-irradiation. Scale bar: 50 μm for immunofluorescence images. (**C**) Intratumoral production of GPX4 activity measurement. Data represent the mean (± standard deviation, SD) of 3 independent experiments.

**Correct Figure 8**:

**Fig. 8:**
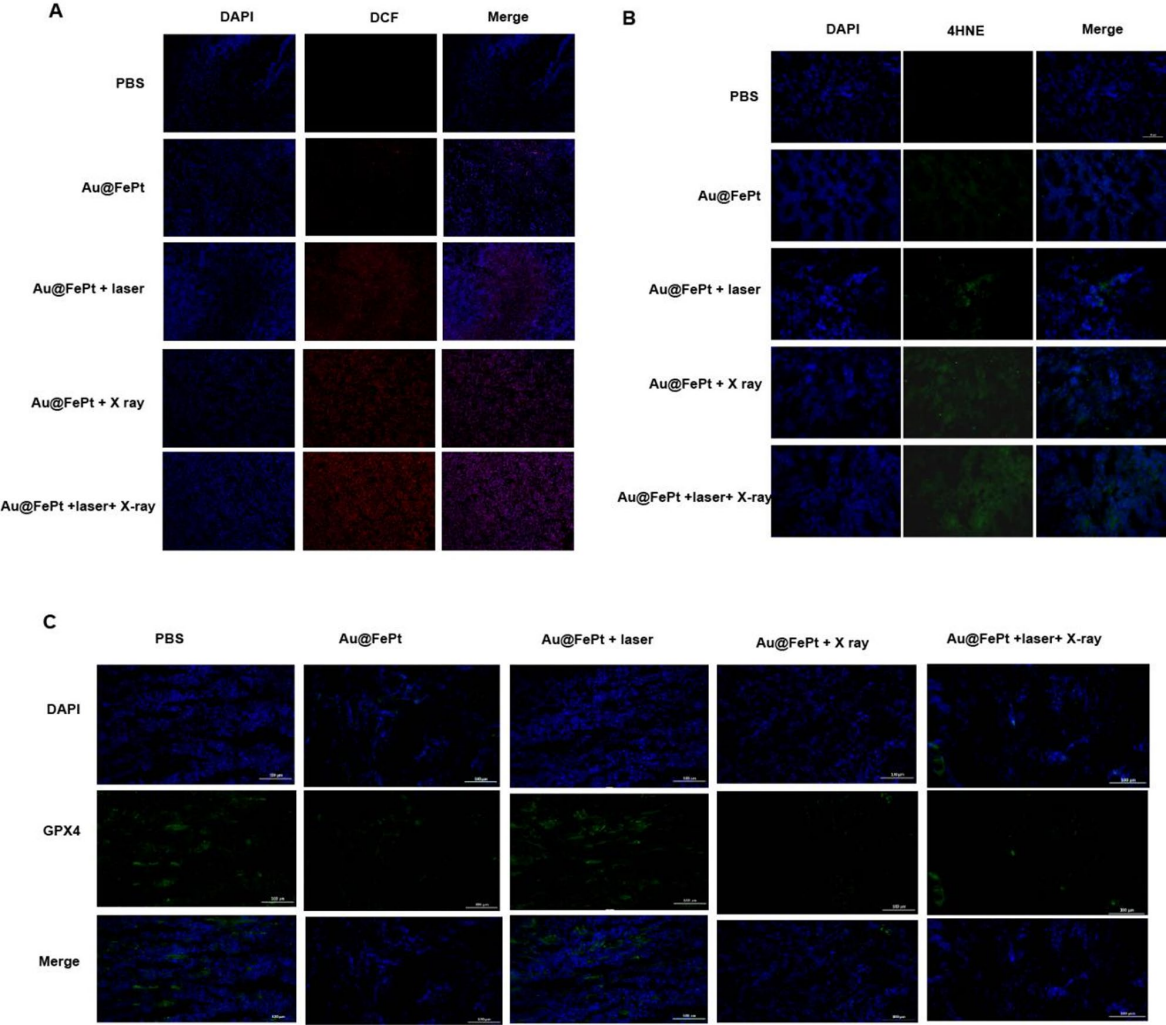
Intratumoral production of (**A**) ROS and (**B**) 4-HNE obtained from tumor tissue slices of mice receiving different treatments group. Mice were sacrificed and tumors were dissected at 24 h post-irradiation. Scale bar: 50 μm for immunofluorescence images. (**C**) Intratumoral production of GPX4 activity measurement. Data represent the mean (± standard deviation, SD) of 3 independent experiments

The original article has been corrected.

