# Supplementary material for: Mild photothermal/radiation therapy potentiates ferroptosis effect for ablation of breast cancer via MRI/PA imaging guided all-in-one strategy
Source: J Nanobiotechnology. 2023 May 8;21:150. doi: 10.1186/s12951-023-01910-6 (PMC10169499; doi:10.1186/s12951-023-01910-6)
Supplement: Supplementary file 1 — Additional file 1. Additional documents. [file 12951_2023_1910_MOESM1_ESM.docx]

Additional file 1

| **Mild Photothermal/Radiation Therapy Potentiates Ferroptosis Effect for Ablation of Breast Cancer *via* MRI/PA Imaging Guided All-in-one Strategy** |
| --- |
| **Zhe Zhang^a^**^‡^**,** **Hsuan Lo^b^**^‡^**, Xingyang Zhao^b^**^‡^**, Wenya Li^b^, Ke Wu^a^, Fanchu Zeng,^b^ Shiying Li^b*^, Hongzan Sun^a*^**  **Affiliations**  a Department of Radiology, Shengjing Hospital of China Medical University, Shenyang, China.  b Guangdong Cardiovascular Institute, Guangdong Provincial People's Hospital, Guangdong Academy of Medical Sciences, Guangzhou 510080, China.  c Medical Research Institute, Guangdong Provincial People's Hospital (Guangdong Academy of Medical Sciences), Southern Medical University, Guangzhou 510080, China.  *** Correspondence Author:**  Shiyng Li, Ph.D.  Guangdong Cardiovascular Institute, Guangdong Provincial People's Hospital, Guangdong Academy of Medical Sciences, Guangzhou 510080, China.  5811sl@gmail.com  Hongzan Sun, Ph.D.  Department of Radiology, Shengjing Hospital of China Medical University, Sanhao Street No. 36, Heping District, Shenyang 110004, China.  sunhongzan@126.com |

**Experimental details:** Chemical and Reagents: Diisobutylamine, phloroglucinol anhydrous, and croconic acid were purchased from Mecklin Scientific Ltd. (Shanghai, China).Dulbecco’s modified Eagle’s medium (DMEM) and FBS were obtained from GIBCO, Ltd., (USA). Methanol (HPLC/Spectro grade, 99.9%), acetonitrile (HPLC grade, 99.9%) and cysteine were obtained from Dieckmann. Ultra-centrifuge filter (MWCO 100 kDa) and dialysis bag (500 kDa) was purchased from Amicon Ultra (Merck Millipore, United States). Antibodies applied for western blot including rabbit anti-actin, and anti- caspase-3 (BD 610959), anti-cleaved PARP-1 (AB4830), anti-cleaved caspase 3 (Immunoway YC0004) were acquired from Abcam. HIF-1 α (BD 610959), CD31 were obtained from BD. CCK-8 kits were purchased from MedChemExpress (Princeton, NJ, USA). Peptide iRGD (Cys–Arg–Gly–Asp–Arg–Gly–Pro–Asp–Cys) (95% purity) was purchased from GR Biochem Ltd (Shanghai, China).

**Synthesis of silver nanoparticles :** The template-engaged galvanic replacement reaction provides a simple easy and reproducible method for preparing hollow nanostructures of noble metals including Au based on silver nanoparticles as templates[1,2]. According to previous literature, gold nanocages (AuNCs) were prepared by reducing HAuCl_4_ on the silver nanoparticle framework with some modifications[3–5]. Silver nanoparticles were first synthesized by stirring silver nitrate solution (0.1 M, 50 ml) with sodium citrate (0.5 M, 1.5 ml) and freshly prepared sodium borohydride solution (0.1 M,1.5 ml). The resulting solution showed a characteristic yellow color immediately upon the addition of NaBH_4_, and the mixture was stirred for at least 3 hrs to complete the reaction. Then hydroxylamine hydrochloride solution (0.2 M, 1.5 ml) was added to silver nanoparticles (50 ml) solution and stirred for ten minutes. Afterward, silver nitrate solution (0.1 M, 1.5 ml) was added, and the mixture was stirred at room temperature overnight. The color of the solution would turn orange.

**Preparation of AuNCs and surface** **modification:** AuNCs were synthesized via the galvanic replacement reaction with silver nanoparticles as the templates. Briefly, silver nanoparticles were firstly synthesized by stirring silver nitrate solution (0.1 M, 50 ml) with sodium citrate (0.5 M, 1.5 ml) and freshly prepared sodium borohydride solution (0.1 M,1.5 ml). The resulting solution showed a characteristic yellow color immediately upon adding NaBH4, and the mixture was stirred for at least 3 hrs to complete the reaction. Then hydroxylamine hydrochloride solution (0.2 M, 1.5 ml) was added to silver nanoparticles (50 ml) solution and stirred for ten minutes. Afterward, silver nitrate solution (0.1 M, 1.5 ml) was added, and the mixture was stirred at room temperature overnight. The color of the solution would turn to orange. AuNCs can be conveniently obtained by simply titrating with 12 ml of (1mM) HAuCl_4_ solution under heat and refluxed for one hour. They were first modified by adding SH-PEG_2000-_NH_2_ (1mg per 50 ml AuNCs solution, LayBio) and stirring for 24 hrs to obtain NH2-PEG@AuNCs. NH2-PEG@Au@FePt was modified *via* similar way.

Then, NH_2_-PEG@AuNCs and NH_2_-PEG@Au@FePt NPs were conjugated with the iRGD peptide through the interaction between EDC/NHS reaction with iRGD peptide for 24 hrs at room temperature in the dark. The molar ratio of Mal-PEG-PLGA NPs and iRGD used for conjugation was 4:1. The resulting solution was transferred into ultrafiltration (MWCO 100 kDa) and centrifuge at 3000 rpm for 15 min to obtain the targeted nanoprobe iRGD-Au@FePt and iRGD-AuNCs NPs. The galvanic replacement reaction will occur when silver nanoparticles are titrated with HAuCl_4_ aqueous solution. In theory, AuCl_4_^−^ oxidizes silver nanoparticles to AgCl attributed to the standard reduction potential of the AuCl_4_^−^ /Au (0.99 V) higher than AgCl/Ag (0.22 V). Au atoms can grow on the surface of the Ag template according to the shared face-centered cubic structure.

**Preparation of iRGD-PEG-PLGA/AuNCs@FePt NPs (Au@FePt) :** They were first modified by the addition of SH-PEG2000-NH_2_ (1mg per 50 ml Au@FePt solution, LayBio) and stirring for 24 hrs to obtain PEG@Au@FePt. The molar ratio of SH-PEG2000-NH_2_ NPs and iRGD used for conjugation was 4:1. The resulting solution was transferred into ultrafiltration (MWCO 30 kDa) and centrifuge at 3000 rpm for 15 min to obtain the targeted nanoprobe iRGD-AuNCs@FePt (Au@FePt) NPs. For control, the PEG-AuNCs@FePt (CAu@FePt) NPs were prepared using the similar protocol mentioned above except without iRGD conjugation.

**Fe release of the Au@FePt nanoprobes** **:**Au@FePt nanoprobes containing 1 mg Fe were dispersed in 1 mL HNO_3_ solution (pH 5.8, 7.4 with and without laser) in dialysis tubing (MWCO = 1 kDa, Spectrum Laboratories, Inc.) and the filtrates were collected for Fe concentration quantification with ICP-OES with 24-hour intervals.

***Measurements of Photothermal Conversion Efficiency of Au@FePt NPs****:* Besides, the laser power density-dependent photothermal conversion was also investigated for continuous adjustment from 0.8 W /cm^2^ to 1.2 W /cm^2^, Furthermore, the photothermal stability of Au@FePt NPs at a concentration of 50 ppm was also measured. To measure the photothermal performance, the photothermal conversion efficacy (η) was calculated according to the Equation 1 and 2 as below. Three test were repeated[6,7].Equation S1

$\eta\mathbf{=}\frac{h_{A}\Delta T_{\max}-Q_{s}}{I\left( 1-10^{-A_{\lambda}} \right)}$ (1)

The value of $hA$ is calculated by the following Equation S2

$hA=\frac{m_{D}C_{D}}{\tau_{s}}$ (2)

where $\boldsymbol{\tau}_{s}$ is the time constant of the sample system, $m_{D}$ and $C_{D}$ are the mass (0.5 g) and heat capacity 2.1 J g^-1^of deionized water when used as the solvent, respectively. In order to get the value of *h*_A_, herein introduce $\theta$, which is defined as the ration of $\Delta Tto\Delta Tmax$_._

The photothermal conversion efficiency ($\eta$)

$$\eta=\frac{h*A*\Delta T-Q_{s}}{I*\left( 1-{10}^{-A_{\lambda}} \right)}=\frac{\left( 0.0081*55.8 \right)-0.0252}{1.5*\left( 1-{10}^{-1.0398} \right)}=31.3\%$$

Find （$h*A$）first

Use $\theta=\frac{T_{\max}-T}{\Delta T}$

By integrating

$$t=\frac{mC}{h*A}*(-\ln\theta)$$

As linear equation

which $m$ is mass, $C$ is heat capacity

$$m=0.5g$$

$$C=2.1J/g$$

***Identification of differentially expressed genes (DEGs) :*** The R package, edge R, was used to identify the DEGs between tumor and normal samples The empirical probability distribution of the fold-changes correlated with significant DEGs was used to define a |log_2_ Fold Change| with a threshold >1.5 and false discovery rate (FDR) < 0.05.

***Annexin V-FITC/PI double staining (flow cytometry)****:* 4T1 were seeded in 6-well plate and incubated with either AuNCs and Au@FePt NPs (100 μg/ml of Fe) for 24 hrs. The cells were collected and washed with PBS three times then resuspended in 100 µl 1X annexin binding buffer. Harvested cells were counterstained with 100 µg/ml propidium iodide (PI) dye and FITC labelled annexin V for 15 mins, and the stained cells were subjected to flow cytometry analysis.

The apoptosis rate = (the number of early apoptotic cells + the number of late apoptotic cells) / total cell count × 100%

***Western blot analysis****:* The apoptosis induced relative protein expression level via FePt, AuNCs or Au@FePt NPs was determined by the western blot analysis. 4T1 cells were treated with FePt, AuNCs or Au@FePt NPs NPs (100 ug/mL of Fe) upon laser irradiation. PBS group was taken as control. After 24 hrs treatment, the protein was extracted from the cells using lysis buffer, separated on SDS-PAGE (Bio-Rad), and quantified using Bradford reagent. Expressed proteins (40 µg each) were transferred to PVDF membrane (Blocking 5 % BSA in TBST) and incubated with various primary antibodies, caspase-3 (BD 610959), anti-cleaved PARP-1 (AB4830), anti-cleaved caspase 3 (Immunoway YC0004) , shake together in the 4 ^o^ C for overnight. The membrane was washed with 1X TBST and added with conjugated secondary antibody for 2 hrs in dark as the reported protocol.[8] The electrochemiluminescence (ECL) assay was used to determine levels of relative proteins.

***Photothermal stability:*** To test the photothermal effect, AuNCs and Au@FePt were exposed to 1064 nm laser (1.2 W/cm^2^, 5 min). During laser irradiation, the temperature was continuously monitored with a thermal camera. To study photothermal stability, the temperature curve of AuNCs and Au@FePt were evaluated before and after being treated upon laser irradiation.

***In Vivo* PAI：**The Vevo LAZR software demonstrated spectra unmixing according to the photoacoustic spectra for the Au@FePt (as described from the imaging of nanoprobe in the tissue-mimicking phantom). Dispersions of Au@FePt nanoprobes at gradient concentrations were used for the evaluation of in vitro imaging performance under different modalities. For PAI, the samples (0, 5, 10, 20, 40, and 100 μg/mL) were tested using two-dimensional photoacoustic image (PA mode) and two-dimensional ultrasound image (B mode) images were acquired with a Vevo LAZR Photoacoustic Imaging System (Vevo VisualSonics). The signal distribution of oxy- and deoxy-hemoglobin in blood was obtained, multiplexed and overlaid on the ultrasound image (B mode). Regions of interest (ROIs) were drawn around the tumor region to receive the resulting PA images for all unmixed components and were quantified via Vevo software in different slides. Image analysis was performed on each slide within the suspected tumor regions. While the effective LZ250 transducer covers the entire tumor, the whole tumor's high spatial and temporal resolution and surrounding areas were imaged by the NIR laser scanning the array in 2D mode. PA multispectral images were recorded at multiwavelengths (680,710,750,800 and 850 nm) to achieve accurate tumor microenvironment mapping.

***Tumor histology and immunohistochemistry:*** After 7 days of treatment, the mice were sacrificed and tumor tissue will collect. Tissue specimens were fixed with 10% neutral-buffered formalin, and 10 μm paraffin sections were then prepared. One section was stained with haematoxylin and eosin (H&E) for histological assessment, and the other sections were immunostained using a streptavidin peroxidase procedure after microwave antigen retrieval. The primary antibodies anti-HIF-1α (1 : 200, provided by BD 610959). Specimens were incubated with the biotinylated secondary antibody against the corresponding primary antibody. Visualisation was performed using diaminobenzidine (DAB). All immunostained sections were then lightly counterstained with Mayer's haematoxylin. Throughout the above analysis, negative controls were prepared by omitting the primary antibody. For HIF-1α staining, we evaluated 500 cells from 10 fields of vision and counted the cells with for each specimen.

***IHC Staining of CD-31 and PCNA:*** Thin sections (10 mm) from formalin-fixed, paraffin-embedded tumor tissues were stained with anti-CD31 (AB182981) antibodies, followed by HRP-conjugated secondary antibody hybridizing. The targeted proteins were visualized with Dako’s Envision system. Besides, similar protocol was preformed, 10µm-thick sections covering the entire tumor volume were extracted. Immunohistochemistry was performed on the frozen sections using antibodies against PCNA.

***Terminal Deoxynucleotidyl Transferase-mediated dUTP Nick-end Labelling (TUNEL) assay:*** The sections on the slides were incubated in fixative at room temperature for 20 minutes, washed twice with PBS for 15 minutes followed by incubation with TUNEL staining mixture (In Situ Cell Death Detection Kit, TMR red, Sevicebio Technology CO LTD) at 37°C for 1 hour. The TUNEL stained slides were visualized using observed using a fluorescence microscope. For TUNEL positive nuclei, the co-localized green stain signals were counted. Whereas, the total number of nuclei was determined by counting blue signals for nuclei staining via DAPI. Finally, the ratio of TUNEL was calculated by the number of apoptotic events counted/total number of cells.

***For in vivo 4-HNE and GPX4 antibody staining:*** the cryosections of tumor tissues were fixed with 100% methanol for 10 min and permeabilized with 0.1%, Triton X-100/3% BSA for 10 min. Slides were then washed three times with DPBS (5 min each) and immersed in blocking buffer (0.3% Triton X-100/5% normal serum in 1× DPBS) for 60 min. Tissues slides were covered with anti-4-HNE and GPX4 primary antibody in dilution buffer (1:25 ratio, 0.3% Triton X-100/1% BSA in 1× DPBS) and left overnight at 4 °C. Afterward, slides were rinsed three times with 1× DPBS for 5 min each; the specimen was incubated with Alexa-488 fluorochrome-conjugate secondary antibody in a dilution buffer (1:200 ratio) for 3 h at room temperature in the dark. Slides were rinsed three times in 1× DPBS for 5 min each, stained with DAPI for 10 min, and covered with Prolong Gold Antifade Reagent for fluorescence microscopy imaging.

***Histopathological Evaluation of Organ Tissues****:* After 21 days of treatment, the mice were sacrificed and main organ tissues (heart, liver, spleen and kidney) were collected for pathology analysis using the same tissue dissection process, and later subjected to hematoxylin and eosin (H&E) staining.

**Biosafety test and blood analysis:**

To further in vivo toxicity study of nanoparticles of Au@FePt, animals were sacrificed at the same time after treatment, and 1 ml blood was collected from the heart. It was composed in tubes containing 20 mg/ml EDTA. Serum was collected from blood after centrifuged at 1500 rpm for 10 min for different biochemical assays, such as blood glucose (GLU), blood urea nitrogen (BUN), low-density lipoprotein cholesterol (LDL-C), and aspartate aminotransferase (AST), High plasma uric acid (UA). Healthy mice were intravenously administered with a single dose of Au@FePt, and three mice received intravenous injection with PBS as a control group. Over 14 days period, the mice were observed for behavior changes, and body weight was monitored.

**Result**


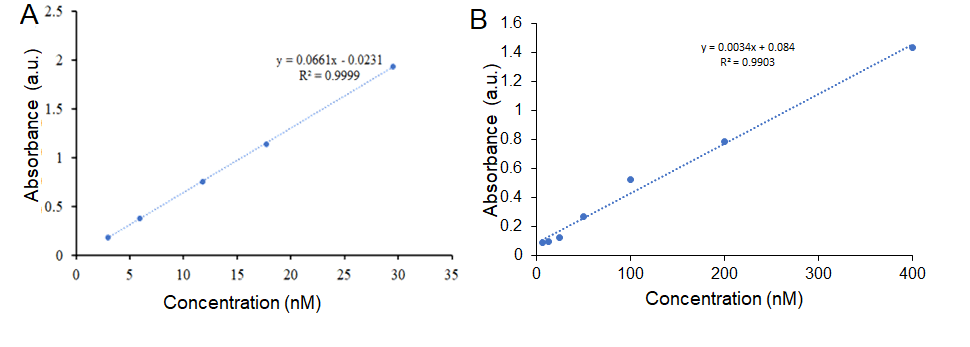


**Figure S1.** (A and B) Excitation spectra of AuNCs and Au@FePt.


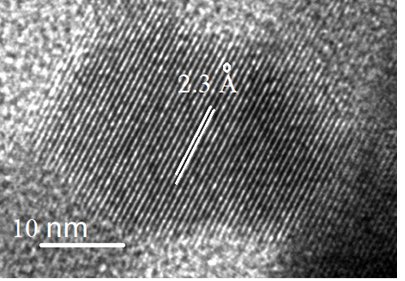


**Figure S2**. (A) HRTEM images of AuNCs NPs. Scale bar 10 nm.


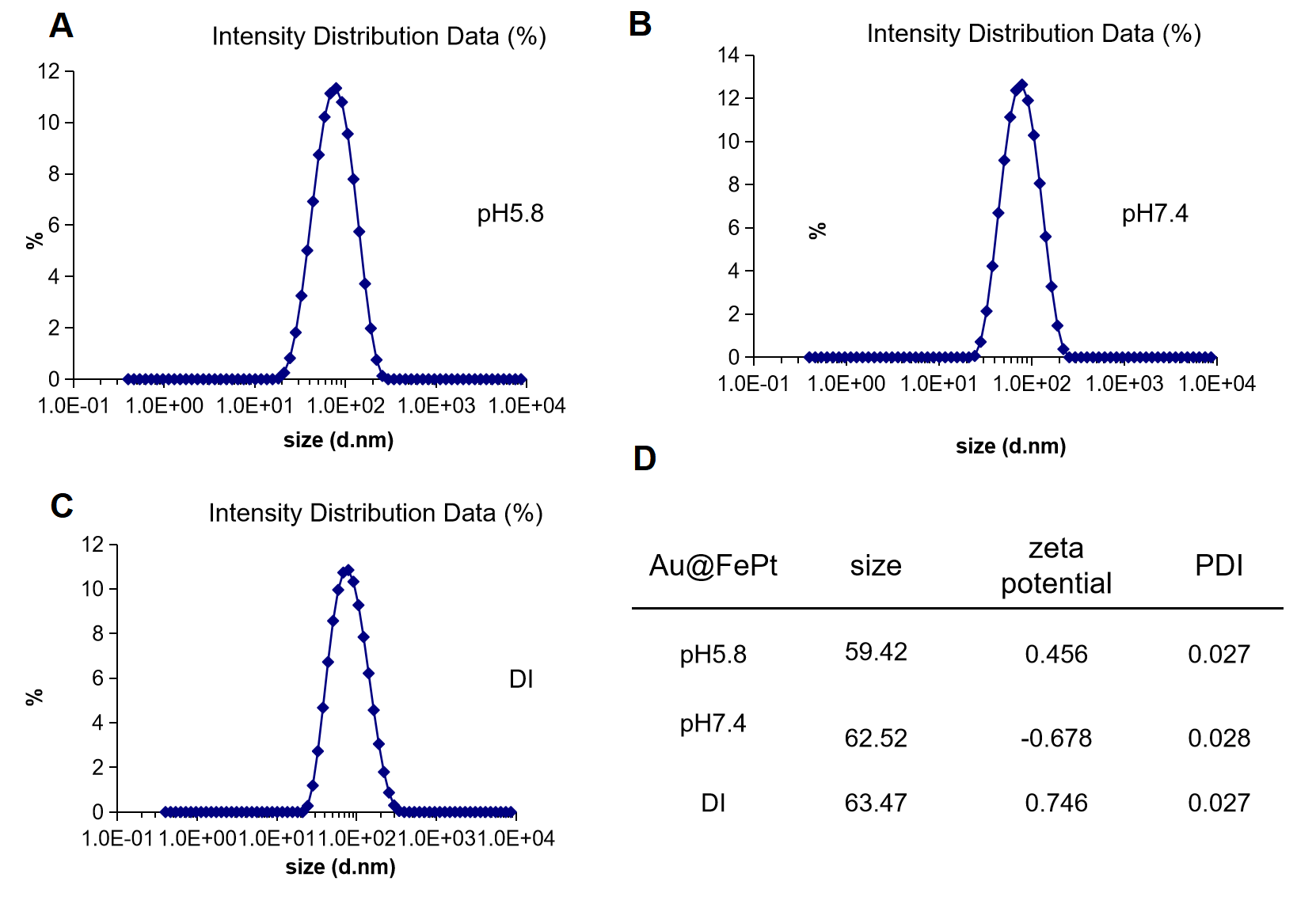


**Figure S3.** DLS size change of Au@FePt nanoprobe at 24 hrs in different buffer solution, a pH 5.8, b pH 7.4, c DI, d summary table.


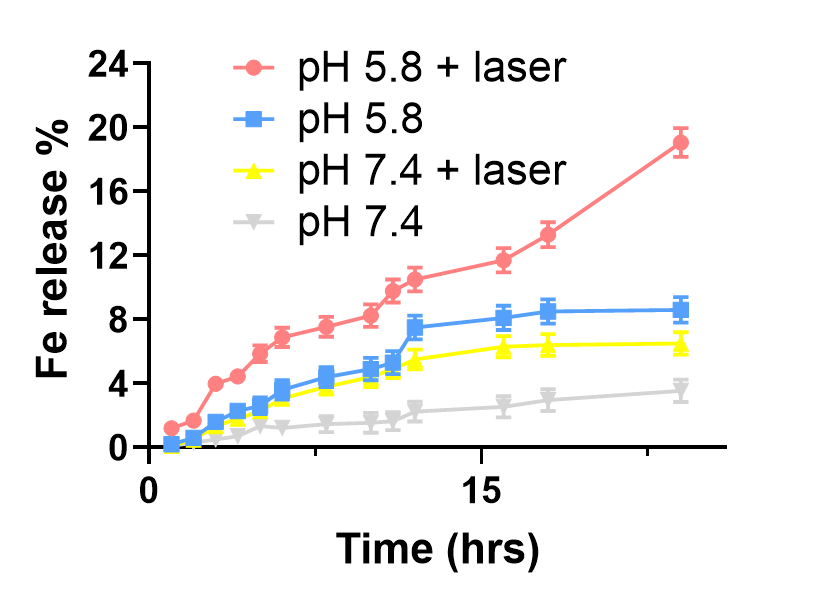


**Figure S4.** Fe release of Au@FePt nanoparticles under various pH with and without laser for 24 h.


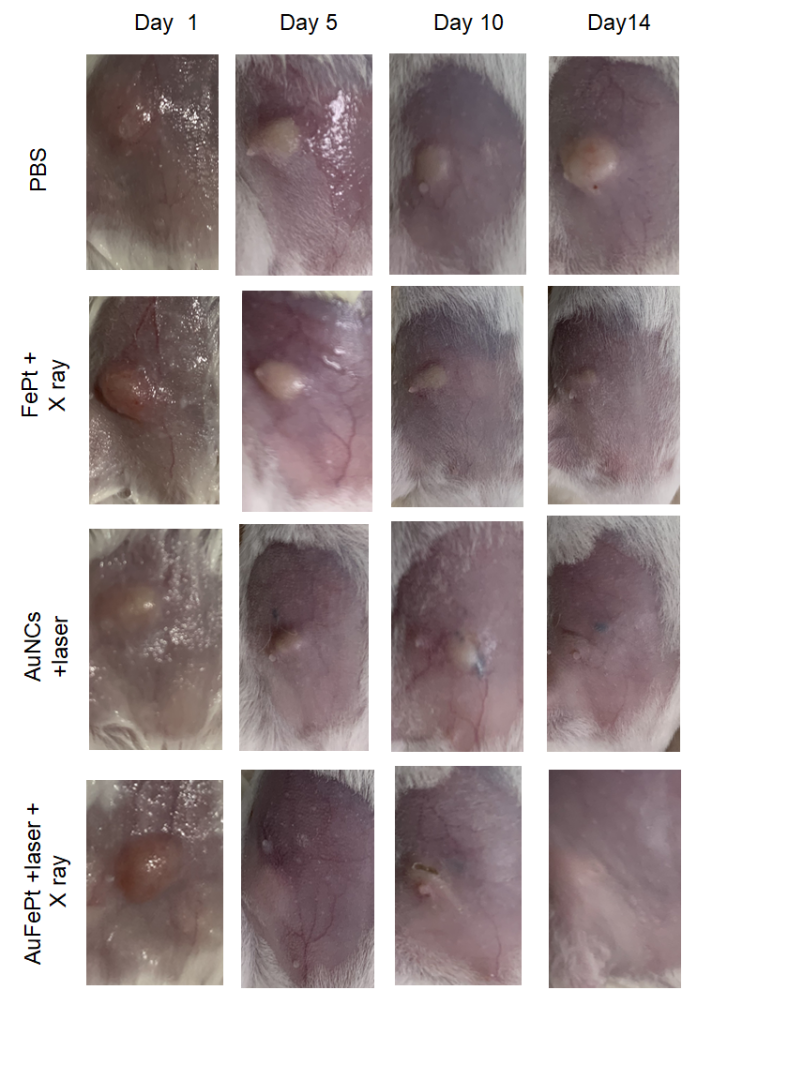


**Figure S5.** (A) Photographs of tumor site for 4T1 tumor-bearing mice post-injection of PBS, FePt +X-ray and AuNCs + laser (1.2 W/cm^2^), Au@FePt + laser + X-ray (n =4).


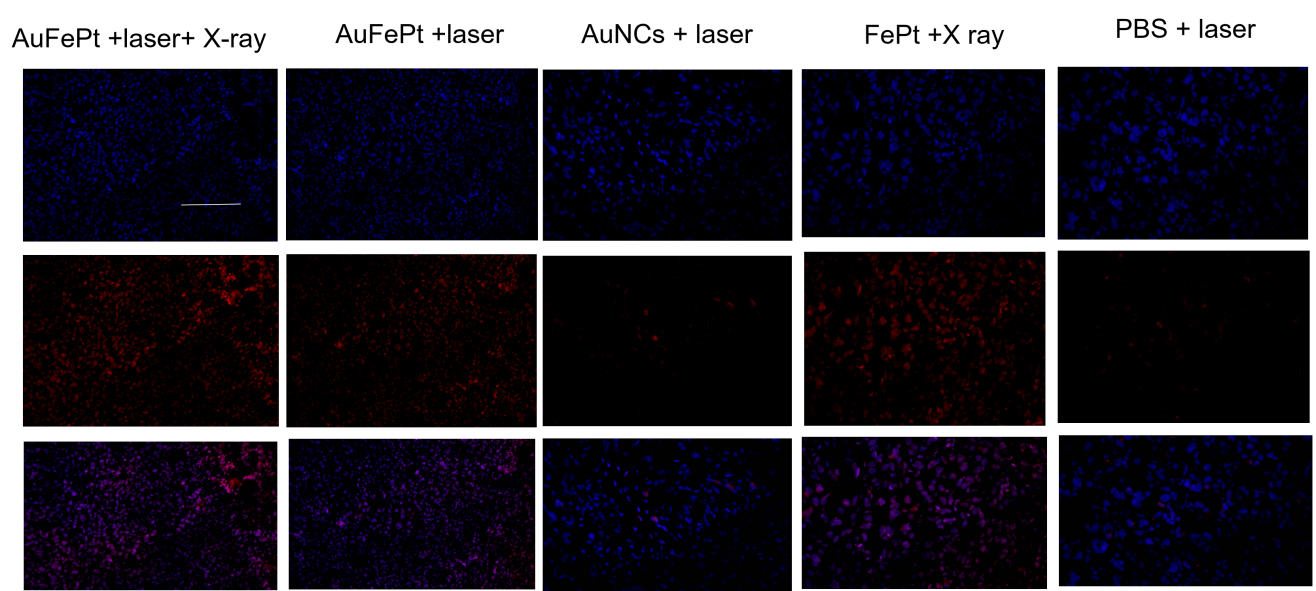


**Figure S6.** Intratumoral production of ROS and from tumor tissue slices of mice receiving different treatments.


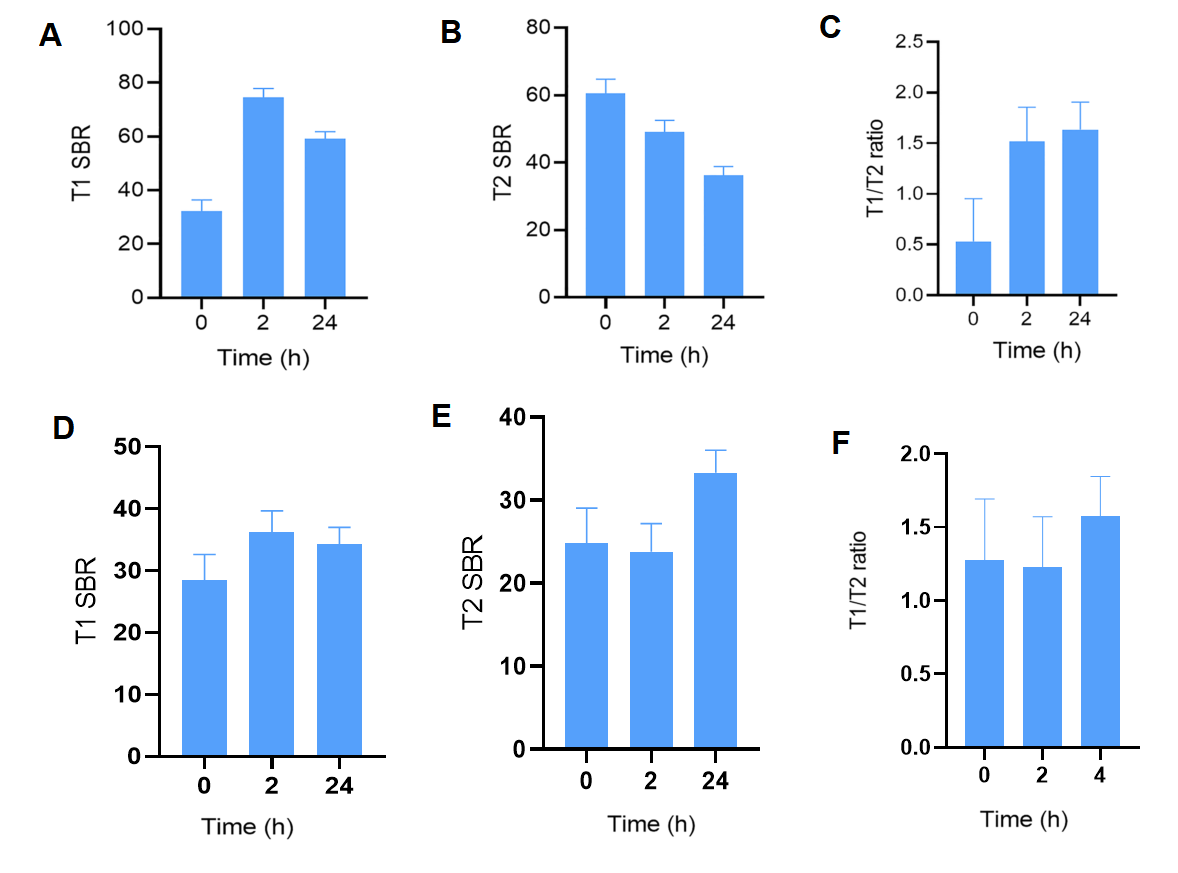


**Figure S7.** (A and B) The corresponding T1 signal to background ratio (SBRs) and T2 SBRs of the tumor area after intravenous injection of Au@FePt nanoprobe at different time points. (C) Ratios of T1 SBR to T2 SBR after intravenous injection of Au@FePt nanoprobe at different time points. (D and E) The corresponding T1 signal to background ratio (SBRs) and T2 SBRs of the tumor area after intravenous injection of FePt nanoprobe at different time points. (F) Ratios of T1 SBR to T2 SBR after intravenous injection of Au@FePt nanoprobe at different time points.


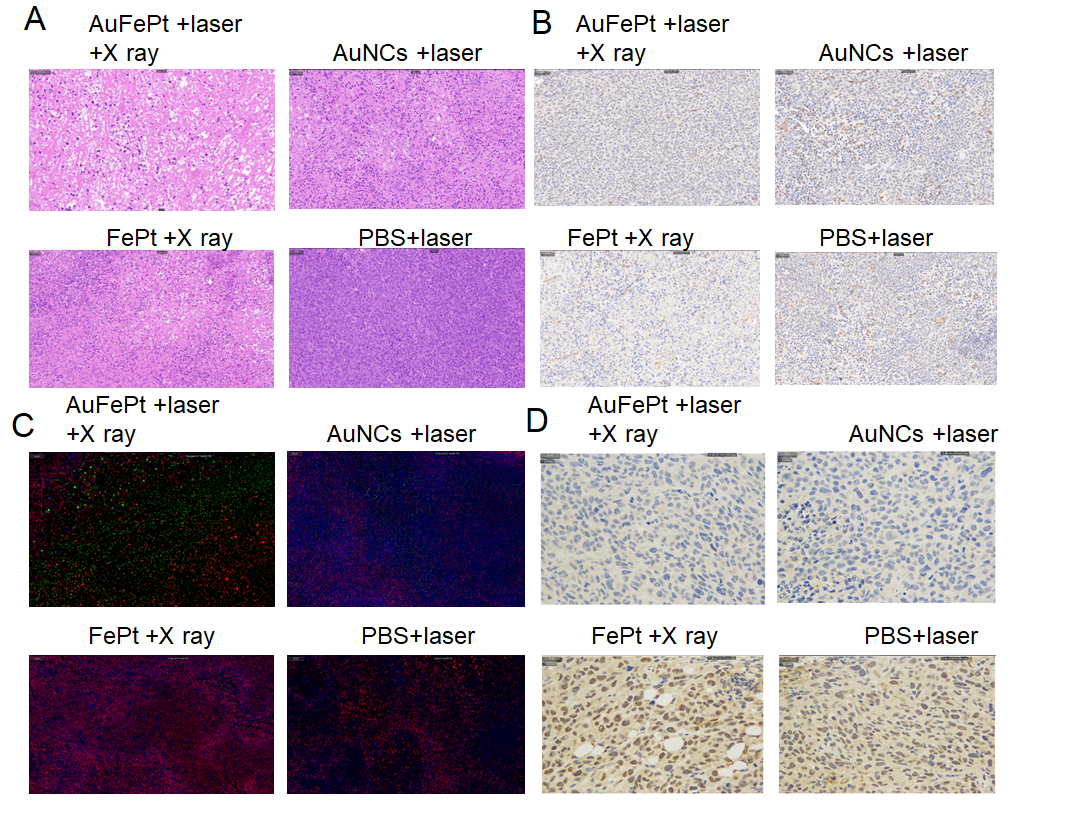


**Figure S8.** (A) The *in vivo* H&E staining results for mice bearing 4T1 orthotopic tumor of PBS+ laser, FePt + X ray, AuNCs + laser, Au@FePt + laser + X ray acquired at 14 days (n=5). The scale bars are 50 μm. (B) IHC staining of abnormal vessels via anti-CD31 antibody; The IHC staining of HIF-1α via anti- HIF-1α antibody. (C) TUNEL assays staining of the different treatment groups, PBS+ laser, FePt + X ray, AuNCs + laser, Au@FePt + laser + X ray acquired at 20 days (n=5). The scale bars are 50 μm. (D) The IHC staining of PCNA via anti-PCNA for tumor of PBS+ laser, FePt + X-ray, AuNCs + laser, Au@FePt + laser + X ray acquired at 20 days (n=5). The scale bars are 50 μm;


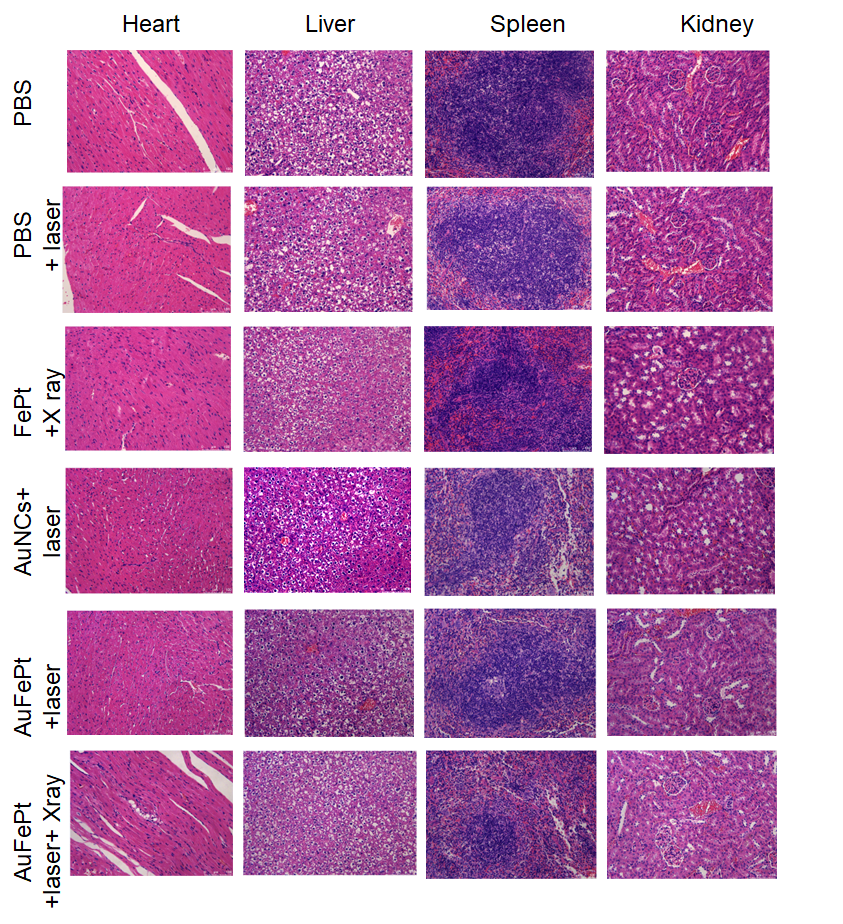


**Figure S9.** H&E staining of main organs of 4T1 tumor-bearing mice post-injection of PBS, FePt and AuNCs, Au@FePt with and without laser irradiation (1.2 W/cm^2^) or X ray after 21 days treatment (n=4). Inserts are the photographs of the relevant scale bar originated from each group. Scale bar 50 mm.


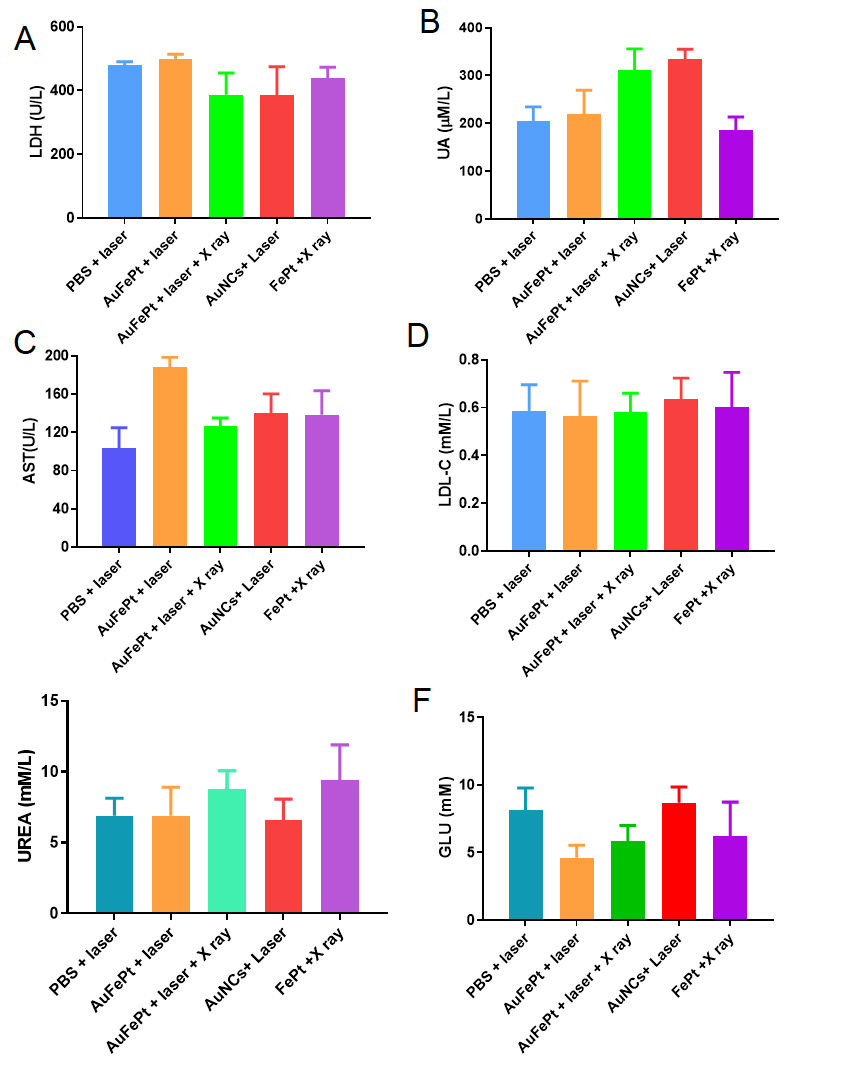


**Figure S10.** The blood biochemical indexes of blood glucose (GLU), blood urea nitrogen (BUN), low-density lipoprotein cholesterol (LDL-C), and aspartate aminotransferase (AST), High plasma uric acid (UA) and lactic dehydrogenase (LDH) which were acquired at 21 days of various treated groups.

**Reference**

1. Li W, Brown PK, Wang L V., Xia Y. Gold nanocages as contrast agents for photoacoustic imaging. Contrast Media Mol Imaging. 2011;6:370–7.

2. Liu C, Li S, Gu Y, Xiong H, Wong W, Sun L. Multispectral Photoacoustic Imaging of Tumor Protease Activity with a Gold Nanocage-Based Activatable Probe. Mol Imaging Biol. 2018;20:919–29.

3. Chen J, Saeki F, Wiley BJ, Cang H, Cobb MJ, Li Z-Y, et al. Gold Nanocages: Bioconjugation and Their Potential Use as Optical Imaging Contrast Agents. Nano Lett. 2005;5:473–7.

4. Skrabalak SE, Chen J, Au L, Lu X, Li X, Xia Y. Gold Nanocages for Biomedical Applications. Adv Mater. 2007;19:3177–84.

5. Li W, Brown PK, Wang L V., Xia Y. Gold nanocages as contrast agents for photoacoustic imaging. Contrast Media Mol Imaging. 2011;6:370–7.

6. Li S, Lui K-H, Li X, Fang X, Lo W-S, Gu Y-J, et al. pH-Triggered Poly(ethylene glycol)–Poly(lactic acid/glycolic acid)/Croconaine Nanoparticles-Assisted Multiplexed Photoacoustic Imaging and Enhanced Photothermal Cancer Therapy. ACS Appl Bio Mater. 2021;4:4152–64.

7. Fang X, Wu X, Li Z, Jiang L, Lo W, Chen G, et al. Biomimetic Anti‐PD‐1 Peptide‐Loaded 2D FePSe 3 Nanosheets for Efficient Photothermal and Enhanced Immune Therapy with Multimodal MR/PA/Thermal Imaging. Adv Sci. 2021;8:2003041.

8. Kazi J, Sen R, Ganguly S, Jha T, Ganguly S, Chatterjee Debnath M. Folate decorated epigallocatechin-3-gallate (EGCG) loaded PLGA nanoparticles; in-vitro and in-vivo targeting efficacy against MDA-MB-231 tumor xenograft. Int J Pharm. 2020;585:119449.
